# Recognition and Resistance in Early Psychotherapeutic Encounters: Therapist Response Style, Narcissistic Admiration and Rivalry, and Public Mental Health Engagement

**DOI:** 10.3390/ijerph23070876

**Published:** 2026-07-05

**Authors:** Avi Besser, Virgil Zeigler-Hill

**Affiliations:** 1 Department of Communication Disorders, Jerusalem Multidisciplinary College, Jerusalem 91010, Israel; 2 Department of Psychology, Oakland University, Rochester, MI 48309, USA; zeiglerh@oakland.edu

**Keywords:** public mental health engagement, mental health service engagement, psychotherapy engagement, mental health promotion, treatment uptake, early retention, trust in services, therapeutic alliance, therapist response style, perceived recognition, autonomy-related resistance, psychological reactance, narcissistic admiration, narcissistic rivalry, treatment expectations

## Abstract

**Highlights:**

**Public health relevance—How does this work relate to a public health issue?**

- Mental health systems face a major public health challenge in translating service availability into actual treatment uptake, trust, early engagement, and retention.
- Early encounters with psychological care may influence whether people perceive mental health services as respectful, credible, autonomy-supportive, and worth continuing.

**Public health significance—Why is this work of significance to public health?**

- In a large community-based vignette experiment, therapist response style shaped proximal engagement-related appraisals, including perceived recognition, autonomy-related resistance, anticipated alliance, credibility, expected benefit, and willingness to continue.
- The findings identify perceived recognition and autonomy-related resistance as potentially modifiable appraisal pathways through which first-contact communication may influence early engagement with psychological care.

**Public health implications—What are the key implications or messages for practitioners, policy makers and/or researchers in public health?**

- Public mental health services, training programs, and intake systems may benefit from emphasizing communication that conveys recognition, respect, and autonomy support during first contacts with potential service users.
- Future public health research should test whether recognition-supportive and autonomy-supportive communication can improve help-seeking, treatment uptake, early retention, and trust in mental health services in real-world clinical and service settings.

**Abstract:**

Early engagement with psychotherapy is a public mental health issue because potential patients’ first appraisals of psychological care may shape treatment expectations, willingness to continue, and openness to receiving effective support. In first-contact therapeutic encounters, people respond not only to the content of a therapist’s intervention but also to the interpersonal meaning conveyed by the therapist’s response style. Guided by a recognition–resistance framework and models of narcissistic self-regulation, we examined how therapist response style and trait narcissistic admiration and rivalry shape early appraisals of psychological care in a vignette-based psychotherapeutic encounter. In a between-subjects vignette experiment, Hebrew-speaking adults in Israel (N = 972) were randomly assigned to read a validation-based, recognition-supportive, autonomy-supportive therapist response or a more directive and challenging response to the same clinical scenario. Participants then reported perceived recognition, autonomy-related resistance, anticipated alliance, therapist credibility, expected benefit, and willingness to continue. The validation-based response elicited higher perceived recognition, lower autonomy-related resistance, and greater willingness to continue. Perceived recognition and autonomy-related resistance mediated the effects of response style on all therapy-related outcomes. Narcissistic admiration predicted more favorable appraisals, and narcissistic rivalry predicted lower recognition and greater resistance, but neither moderated style effects nor indirect pathways. Recognition and autonomy-related resistance emerged as proximal appraisal pathways linking therapist response style to anticipated engagement with psychological care in this analogue vignette context. However, the predicted moderation and moderated-mediation effects involving narcissistic admiration and rivalry were not supported. This pattern suggests that, in the present design, admiration and rivalry functioned more as general appraisal orientations than as differential-susceptibility moderators of therapist response style. The moderated-mediation component of the recognition–resistance framework should therefore be regarded as unsupported pending independent replication and more ecologically valid tests. These findings position first-contact therapist communication as a candidate modifiable feature of public mental health engagement, with implications for future research on treatment uptake, early retention, trust in services, and access to effective psychological care.

## 1. Introduction

Psychotherapy is fundamentally interpersonal. Clients respond not only to the technical content of a therapist’s intervention but also to the way that intervention is delivered and the interpersonal message it communicates. Early therapist responses may convey understanding, validation, recognition, and respect for autonomy, or they may be experienced as more directive, corrective, or challenging. These interpersonal differences can shape how clients evaluate the therapist, the anticipated therapeutic relationship, and the potential value of the treatment. However, therapist responses are unlikely to affect all individuals in the same way. Drawing on contemporary models of narcissistic self-regulation, the present study examined whether narcissistic admiration and narcissistic rivalry help explain why the same therapist behavior may be experienced as affirming and engaging by some individuals but as threatening, autonomy-limiting, or resistance-provoking by others.

This focus on the interpersonal meaning of therapist behavior is consistent with the broader literature on evidence-based psychotherapy relationships and therapist responsiveness, which emphasizes that treatment effectiveness depends not only on the techniques therapists use but also on how those techniques are adapted to the characteristics, needs, and perceptions of individual clients [1]. Indeed, a substantial body of meta-analytic evidence indicates that relational factors such as therapeutic alliance, empathy, and positive regard are among the most robust predictors of psychotherapy outcomes [2,3,4]. Understanding how clients interpret therapist behavior may therefore provide important insight into the psychological processes through which therapeutic relationships develop and influence treatment engagement.

These issues also have important implications for public mental health. A major challenge facing mental health systems is not simply providing access to services but ensuring that individuals are willing to seek care, engage with treatment, and remain involved long enough to benefit from it. Many individuals who could benefit from psychological services either never initiate treatment or discontinue care prematurely. As a result, factors that influence early engagement, trust, and willingness to continue treatment are of considerable public health significance. Initial interactions with mental health providers may represent a particularly important point in this process because they can shape whether individuals perceive psychological care as credible, respectful, helpful, and worth pursuing.

From this perspective, therapist response style can be viewed more cautiously as a candidate modifiable feature of early mental health service engagement. Communications that foster recognition, autonomy, and psychological safety may support anticipated engagement, whereas responses experienced as controlling or autonomy-threatening may undermine it. Examining such appraisals may therefore contribute to public mental health research on help-seeking, early engagement, continuity of care, and access to effective psychological services.

### 1.1. Therapist Response Style and the Therapeutic Relationship

Psychotherapy outcomes depend not only on treatment models but also on the quality of the therapeutic relationship and on how therapists respond to patients in session. A major meta-analysis of adult psychotherapy found a robust positive association between therapeutic alliance and treatment outcome across treatment formats and theoretical orientations [2]. Complementing this, meta-analytic work has shown that therapist empathy is a moderately strong predictor of client outcome [3], and that therapist positive regard is also reliably associated with better treatment results [4]. Taken together, patients tend to benefit when they experience therapists as collaborative, understanding, and accepting.

These relational effects can be shaped by relatively brief therapist behaviors. In an analogue experiment, it was found that therapists’ warmth and competence increased positive outcome expectations and alliance [5]. A systematic review likewise concluded that therapists’ verbal interventions are meaningfully related to the therapeutic alliance and may also have implications for patient outcome [6]. Process research also suggests that therapist challenge is a clinically consequential but delicate intervention whose impact may depend on how it is introduced and negotiated within the therapeutic relationship [7]. Therapist response style is therefore not a superficial stylistic variation but a psychologically consequential feature of the therapeutic encounter.

These findings have direct implications for vignette-based research, because they support the assumption that participants can form meaningful anticipatory judgments about therapist style from relatively brief materials. Such judgments are not merely superficial impressions. Rather, they may reflect clinically relevant appraisals concerning safety, legitimacy, credibility, and the expected quality of the therapeutic relationship. This makes standardized therapist-response vignettes a useful method for isolating the interpersonal meaning of therapist interventions while holding the patient context constant.

### 1.2. Differential Responses to Therapist Style: Preferences, Expectations, and Reactance

The effects of therapist style are not uniform across patients. One of the clearest demonstrations of differential responding to therapist style comes from the literature on patient coping and reactance. A meta-analytic review found that psychotherapy outcomes are improved when treatment is matched to patients’ reactance level such that highly reactant patients tend to fare better in less directive treatments, whereas patients lower in reactance tend to benefit more from greater therapist directiveness [8].

More broadly, patients differ in the kinds of therapist activities and interpersonal styles they prefer, including the degree of therapist versus client directiveness, emotional intensity versus reserve, and warm support versus focused challenge. The Cooper-Norcross Inventory of Preferences was developed precisely to assess these clinically meaningful differences in what clients want from psychotherapy [9]. Meta-analytic evidence further indicates that alignment with client preferences is associated with greater treatment satisfaction, better completion, and modestly better clinical outcomes [10,11]. Taken together, these findings suggest that the same therapist behavior may be experienced as helpful guidance by one individual but as overly confrontational or controlling by another.

A related reason to expect differential responding concerns treatment expectations. Patients’ early expectations about treatment outcome are positively associated with posttreatment outcomes [12]. Immediate responses to a therapist may therefore matter not only as momentary impressions but also because they shape early expectations concerning whether the therapist is likely to be helpful, credible, and worth continuing with. Accordingly, anticipated therapeutic alliance, therapist credibility, expected treatment benefit, and willingness to continue may be understood as early indicators of how participants construe the therapeutic situation.

Reactance theory offers a further conceptual lens for understanding such differential responses. When an interpersonal message is experienced as threatening personal freedom, it may evoke anger, resistance, and oppositional cognition rather than engagement [13]. Applied to psychotherapy, this framework suggests that therapist responses that feel overly interpretive, pressuring, or autonomy-limiting may undermine engagement, particularly among individuals who are more sensitive to interpersonal control. Importantly, feeling understood and feeling controlled should not necessarily be assumed to represent opposite ends of a single continuum. A therapist may be experienced as warm yet somewhat imposing, or as challenging yet still respectful. Accordingly, it is theoretically useful to distinguish recognition-related appraisals from autonomy-related resistance appraisals.

More broadly, psychological reactance has been conceptualized as a motivational response to threatened freedom, involving efforts to restore autonomy when a message is experienced as controlling or pressuring [14]. Meta-analytic evidence further supports the role of reactance in shaping resistance to controlling messages, suggesting that perceived freedom threat is a reliable antecedent of oppositional responding across communication contexts [15].

### 1.3. Narcissism in Psychotherapy

Narcissism is a plausible factor in early psychotherapy engagement because treatment often involves self-evaluation, recognition, vulnerability, dependence, and authority. Although empirical work on narcissism in psychotherapy is more limited than the broader alliance literature, available evidence suggests that narcissistic pathology can complicate treatment entry, engagement, and process. Pathological narcissism has been linked to psychotherapy utilization, initial symptom severity, and early treatment change [16], and narcissism has been associated with dropout from cognitive-behavioral therapy for eating disorders [17]. Clinical scholarship also emphasizes that alliance building is often challenging in the treatment of narcissistic personality disorder because self-esteem regulation, sensitivity to evaluation, and vulnerability to perceived slights may complicate early engagement [18]. Related experimental work suggests that narcissistic pathology is associated with intensified emotional reactions to negative interpersonal events, especially when humiliation or evaluation is salient [19].

Therapist-side research converges with this view. Narcissistic personality disorder has been associated with distinctive countertransference patterns, including more hostile, criticized, helpless, and disengaged therapist responses and less positive therapist response [20]. In addition, research on supportive versus interpretive psychodynamic group therapy indicates that narcissistic pathology and treatment style can be examined empirically within the same therapeutic framework, even if the evidence remains limited and mixed [21]. Taken together, these findings do not imply that narcissism uniformly undermines psychotherapy. Rather, they suggest that early treatment processes may be sensitive to the interpersonal meaning, evaluative tone, and regulatory implications of therapist responses. Because much of the existing treatment literature has focused on pathological narcissism or narcissistic personality disorder, the present study adopts a dimensional lens by distinguishing between narcissistic admiration and narcissistic rivalry.

### 1.4. Narcissistic Admiration and Rivalry as Distinct Self-Regulatory Orientations

The Narcissistic Admiration and Rivalry Concept distinguishes two related but different dimensions of grandiose narcissism [22]. Admiration is more closely tied to agentic self-enhancement, positive visibility, and the pursuit of social approval, whereas rivalry is more closely tied to defensiveness, antagonism, and the protection of status under perceived threat. This distinction is useful for psychotherapy research because therapist responses may communicate understanding, legitimacy, and collaborative recognition, but they may also imply interpretation, authority, pressure, or constraint.

More broadly, narcissism has been conceptualized as a dynamic self-regulatory system organized around the maintenance of desired self-views [23], and narcissistic expressions appear especially sensitive to status-relevant situational cues [24]. Admiration and rivalry are both linked to status pursuit but through different interpersonal orientations: admiration aligns more closely with assertive and self-enhancing routes to status, whereas rivalry aligns more closely with antagonistic and conflictual routes [25]. Rivalry also shows stronger associations with emotion regulation difficulties, reinforcing the expectation that it may be especially relevant to perceptions of threat, defensiveness, and resistance in evaluative interpersonal contexts [26].

Applied to psychotherapy, this framework suggests that therapist response style may function as a psychologically meaningful cue. For individuals higher in admiration, responses that signal understanding, legitimacy, and respect may be especially likely to support recognition-based appraisals. For individuals higher in rivalry, responses that imply evaluative superiority, pressure, or diminished agency may be especially likely to activate autonomy-related resistance. Given the limited clinical evidence and the analogue nature of the present design, these expectations were tested as hypotheses rather than treated as established clinical assumptions.

### 1.5. Recognition and Autonomy-Related Resistance as Appraisal Pathways

The distinction between feeling recognized and experiencing autonomy-related resistance provides a promising way to organize these processes. On the one hand, therapist responses may vary in the degree to which they make the patient feel seen, understood, respected, and treated as legitimate. On the other hand, therapist responses may vary in the degree to which they feel pressuring, interpretively imposing, autonomy-limiting, or freedom-threatening. The literature on therapist directiveness, client preferences, and psychological reactance suggests that these are not identical experiences [6,9,13]. A therapist may be experienced as warm yet still somewhat controlling, or as challenging yet nonetheless respectful. This makes it theoretically useful to model recognition-related appraisals and autonomy-related resistance appraisals as partially distinct pathways rather than as opposite poles of a single dimension.

This distinction is also consistent with broader theory and evidence on reactance, according to which resistance is a motivated response to threatened freedom rather than merely the absence of positive evaluation [14,15]. From this perspective, feeling recognized and feeling constrained should not be treated as simple opposites, because both appraisals may coexist within the same interpersonal encounter. This distinction is also consistent with psychotherapy research grounded in self-determination theory. In brief treatments for depression, patients’ autonomous motivation for therapy predicted better treatment outcome, and was higher when therapists were experienced as more autonomy-supportive [27,28]. These findings strengthen the rationale for treating recognition-related appraisals and autonomy-related resistance appraisals as clinically meaningful pathways in anticipated responses to therapist style.

This dual-pathway view also aligns with our recent work across several socially meaningful contexts. In our study of older adults’ evaluations of empathic versus cold socially assistive robots, perceived recognition emerged as a central mechanism linking narcissistic admiration and rivalry to downstream evaluations, while also showing that greater empathy was not uniformly beneficial for individuals higher in rivalry-related threat sensitivity [29]. In our study of self-relevant advertising, responses were likewise organized around two partially distinct pathways, perceived recognition and autonomy-related resistance, with admiration linked more strongly to recognition-based responding and rivalry showing a more conflicted pattern involving both recognition and resistance [30]. In our study of everyday playground encounters among young parents, recognition-based versus status-challenging norm framing shaped appraisals of perceived recognition and perceived freedom threat, which in turn predicted reactance-related, affective, evaluative, and behavioral outcomes [31]. Taken together, these studies provide preliminary evidence for recognition-related and resistance-related appraisal pathways and offer a rationale for examining these processes in psychotherapy. However, the repeated absence of the predicted moderation effects across these related analogue studies requires caution in treating narcissistic admiration and rivalry as differential-susceptibility moderators of the framework. Unlike human–robot interaction, psychotherapy is a human helping relationship marked by asymmetrical expertise, explicit emotional disclosure, and direct implications for self-understanding and change. Unlike advertising, psychotherapy is not a persuasive communication context organized around consumer choice. Unlike playground interaction, psychotherapy is not a brief peer-level social encounter but a structured helping situation in which authority, dependence, and alliance are central. For these reasons, psychotherapy is not simply another context for the same process. It is a clinically consequential context in which recognition-related appraisals and autonomy-related resistance may have potentially important implications for anticipated alliance, therapist credibility, and willingness to remain engaged.

### 1.6. The Present Study

Against this background, the present study uses a vignette-based design to compare therapist response styles embedded in the same clinical scenario. Specifically, the key contrast is between a validation-based, recognition-supportive, autonomy-supportive therapist response and a more directive and challenging therapist response, while holding the patient context constant. This design allows the interpersonal meaning of therapist response style to be isolated more clearly than would be possible in a broader comparison of therapeutic approaches, while also mapping directly onto the studies on alliance, empathy, challenge, therapist statements, patient reactance, and client preferences.

The present study therefore examines whether narcissistic admiration and rivalry shape individuals’ immediate responses to therapist response style in a vignette-based psychotherapeutic encounter. We propose two central process variables. The first is perceived recognition, defined as the extent to which the participant feels seen, understood, respected, and treated as legitimate by the therapist. The second concerns autonomy-related resistance, operationalized here through perceived freedom threat and state reactance. These appraisal processes are expected to shape downstream evaluations of both the therapist and the therapeutic encounter, including anticipated therapeutic alliance, therapist credibility, expected treatment benefit, and willingness to continue with the therapist. In this sense, the present study examines whether the recognition–resistance framework can be extended to psychotherapeutic encounters, where the relational stakes are especially high [29,30,31]. By focusing on willingness to continue, expected benefit, credibility, and anticipated alliance, the study also addresses early engagement as a public mental health process. These outcomes are relevant to whether individuals are likely to enter, remain in, and collaborate with psychological care, especially when they encounter therapist communication that may either preserve or threaten recognition and autonomy.

**Figure 1:**
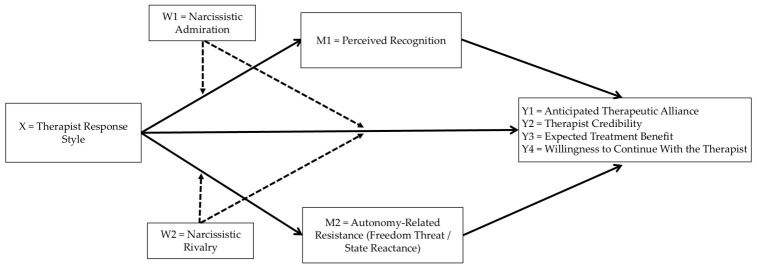
Hypothesized moderated mediation model linking therapist response style, narcissistic admiration and narcissistic rivalry, appraisal processes, and anticipated therapy-related outcomes. Note. Therapist response style (X) was hypothesized to predict perceived recognition and autonomy-related resistance (*M*), operationalized here through perceived freedom threat and state reactance. These appraisal processes were expected to predict anticipated therapeutic alliance, therapist credibility, expected treatment benefit, and willingness to continue with the therapist (Y). Narcissistic admiration and narcissistic rivalry (W) were hypothesized to moderate the effects of therapist response style on the appraisal variables, thereby producing conditional indirect effects. Solid lines indicate hypothesized direct and mediational paths, and dashed lines indicate hypothesized moderating effects.

Figure 1 presents the theoretical model of the hypothesized associations among therapist response style, narcissistic admiration and rivalry, appraisal processes, and anticipated therapy-related outcomes. Therapist response style was hypothesized to predict perceived recognition and autonomy-related resistance, operationalized here through perceived freedom threat and state reactance. These appraisal processes were expected to predict anticipated therapeutic alliance, therapist credibility, expected treatment benefit, and willingness to continue with the therapist. Narcissistic admiration and narcissistic rivalry were expected to moderate the effects of therapist response style on the appraisal variables, thereby shaping the downstream pattern of anticipated therapy-related outcomes.

### 1.7. Hypotheses

Guided by the recognition–resistance framework and by the literature on therapist response style, psychological reactance, and narcissistic self-regulation, we advanced the hypotheses below. These preserve the substantive content of the preregistered hypotheses but are organized to follow the tested appraisal-process model, distinguishing condition effects, mediator–outcome associations, indirect effects, moderation of the appraisal layer, and moderated indirect effects.

**H1.**  *Participants exposed to a validation-based, recognition-supportive, autonomy-supportive therapist response will report higher perceived recognition, lower autonomy-related resistance, stronger anticipated therapeutic alliance, higher therapist credibility, greater expected treatment benefit, and greater willingness to continue with the therapist than participants exposed to a more directive and challenging therapist response.*

**H2.**  *Perceived recognition will be positively associated with anticipated therapeutic alliance, therapist credibility, expected treatment benefit, and willingness to continue with the therapist. In contrast, autonomy-related resistance will be negatively associated with anticipated therapeutic alliance, therapist credibility, expected treatment benefit, and willingness to continue with the therapist.*

**H3.**  *Therapist response style will show indirect associations with each outcome through perceived recognition and autonomy-related resistance. Specifically, the validation-based condition (vs. challenge-based condition) will be associated with anticipated therapeutic alliance, therapist credibility, expected treatment benefit, and willingness to continue with the therapist via higher perceived recognition and via lower autonomy-related resistance.*

**H4.**  *Narcissistic admiration and narcissistic rivalry will moderate the associations between therapist response style and the mediators (i.e., perceived recognition and autonomy-related resistance). We expected admiration to be associated with greater differentiation between conditions in perceived recognition, whereas rivalry was expected to be associated with greater differentiation between conditions in autonomy-related resistance.*

**H5.**  *Narcissistic admiration and narcissistic rivalry will moderate the indirect associations between therapist response style and the outcomes through perceived recognition and autonomy-related resistance. We expected admiration to moderate the indirect associations through perceived recognition, whereas rivalry was expected to moderate the indirect associations through autonomy-related resistance.*

## 2. Materials and Methods

### 2.1. Design and Preregistration

The study employed a preregistered between-subjects vignette experiment with two therapist-response conditions: (a) a validation-based, recognition-supportive, autonomy-supportive response, hereafter referred to as the Validation-Based condition, and (b) a more directive and challenging response, hereafter referred to as the Challenge-Based condition. Participants were randomly assigned in a 1:1 ratio to one of the two conditions within the survey platform after providing informed consent. The study was preregistered prior to data collection on the Open Science Framework (OSF; https://osf.io/asq42, accessed on 1 July 2026), including the hypotheses, eligibility and exclusion criteria, primary measures, and analytic strategy.

#### Clarifications Relevant to the Preregistration

The study followed the preregistered design, primary variables, measures, and central analytic framework. Several clarifications relative to the preregistration should be noted. First, the hypotheses are presented in a reorganized form that preserves the substantive content of the preregistered hypotheses while separating the tested model into condition effects, mediator-outcome associations, indirect effects, moderation of the appraisal layer, and moderated indirect effects. Second, although the preregistration stated that the two attention-check items would be completed at the end of the survey, they were embedded in the questionnaire, one in the first third and one in the final third, to assess attentiveness across the survey. Third, in addition to the preregistered attention-check and invariant-response exclusions, data-quality exclusions for univariate outliers and highly inconsistent response patterns were applied prior to hypothesis testing. Fourth, conditional indirect effects were estimated using 10,000 rather than 5000 bootstrap resamples to increase the stability of confidence interval estimates. Finally, therapist-response condition was coded in the reverse direction relative to the preregistration, with the validation-based response coded +1 and the challenge-based response coded −1; this affects only the sign of coefficients, not their substantive interpretation.

This design was selected to isolate the interpersonal meaning of therapist response style while holding the patient’s presenting problem constant. Compared with a broader comparison of therapy schools, a response-style manipulation afforded tighter experimental control and more direct testing of the proposed appraisal processes. More specifically, by varying therapist response style within the same clinical scenario, the design allowed the psychological meaning of the therapist’s response to be examined while minimizing confounds related to differences in treatment model, clinical context, or presenting problem. The study was specifically designed to test the recognition–resistance framework in the distinct context of early psychotherapeutic encounters rather than to replicate our earlier paradigms in a new setting [29,30,31]. Unlike our prior playground norm-framing study, which focused on young parents responding to everyday community-based parenting encounters, the present study recruited a broad adult sample and examined appraisals of therapist response style, anticipated therapeutic alliance, therapist credibility, expected treatment benefit, and willingness to continue psychological care. Thus, although the studies share a general appraisal-process framework, the present design differs in its target population, interpersonal context, experimental stimulus, and outcome domain.

### 2.2. Participants

A total of 1107 Hebrew-speaking adults in Israel were recruited through iPanel (Shanghai, China), an online survey research company that provides access to a large panel of potential respondents. Study invitations were distributed through the iPanel system to panel members who met the study’s eligibility criteria, and participation was voluntary and compensation was provided in accordance with the panel’s standard practice (10 ILS).

Participants were eligible if they were at least 18 years old, fluent in Hebrew, and able to complete the study via the online survey platform. To preserve the intended participant perspective, individuals who were licensed mental health professionals, psychotherapy trainees, or graduate students in clinical psychology, counseling, psychotherapy, or clinical social work were excluded. These exclusions were intended to reduce the likelihood that respondents would evaluate the vignette primarily from a clinician’s perspective rather than from the perspective of a potential patient. The preregistered target final analyzable sample was 1000 participants, with approximately equal allocation across the two experimental conditions.

Participants were excluded prior to analysis if they were univariate outliers (*n* = 20), had highly inconsistent response patterns indexed by unusually large inter-item standard deviations (*n* = 68), or had evidence of straightlining (i.e., providing the same response across a large number of items) identified via longstring analysis (*n* = 14). In addition, 14 participants in the validation-based condition and 19 participants in the challenge-based condition were excluded for failing the attention-check items. After applying the exclusion criteria, the final analytic sample comprised 972 participants, of whom 490 (50.4%) were women and 482 (49.6%) were men. Participants were randomly assigned to the validation-based condition (*n* = 496; 247 women [49.8%], 249 men [50.2%]) or to the challenge-based condition (*n* = 476; 243 women [51.1%], 233 men [48.9%]). Participants ranged in age from 18 to 84 years (*M* = 45.12, *SD* = 14.90). Descriptive statistics for education, employment status, income, religiosity, family composition, and psychotherapy history are reported in Table 1.

The target sample size was selected to provide adequate power for detecting small condition effects and small regression-based associations in the planned conditional process models. A sensitivity power analysis indicated that, with the final analytic sample of 972 participants allocated approximately equally across the two conditions (*n* = 496 and *n* = 476), a two-tailed independent-samples comparison at α = 0.05 had 80% power to detect an effect of approximately Cohen’s d = 0.18, and approximately 88% power to detect Cohen’s d = 0.20. For regression-based tests of individual paths in the conditional process models, the final sample provided 80% power to detect very small incremental effects of approximately f^2^ = 0.008. Thus, the study was well powered for small main and path effects, although very small interaction or moderated-mediation effects may still require larger or more targeted samples.

### 2.3. Procedure

After providing informed consent electronically, participants completed the study online via the survey platform. Participants first completed a brief background questionnaire assessing demographic characteristics and psychotherapy-related background variables. They were then randomly assigned in a 1:1 ratio within the survey platform to read one of two therapist-response vignettes. The vignette was presented in the second person to facilitate identification with the patient role, and participants were instructed to imagine that they were attending an early psychotherapy session and that the therapist’s response was directed toward them personally.

Immediately after reading the vignette, participants completed the main process and outcome measures. The Narcissistic Admiration and Rivalry Questionnaire was administered after the vignette-based measures to reduce the possibility that explicit self-reflection on narcissistic traits would shape participants’ responses to the therapist stimulus. To avoid contaminating the substantive outcome measures, the two manipulation-check items were presented only after all primary vignette-based questionnaires had been completed. Response attentiveness was assessed using two embedded attention-check items, one placed in the first third of the questionnaire and the other in the final third. The study took approximately 15 min to complete, after which participants were debriefed.

### 2.4. Vignette Development and Experimental Manipulation

#### 2.4.1. Clinical Scenario and Rationale

The experimental stimulus consisted of a written vignette describing an early psychotherapy encounter between a patient and a psychologist. The vignette was developed specifically for the present study in order to capture a first-contact therapeutic situation rather than other sorts of social situations (e.g., an everyday community encounter, consumer message, human-technology interaction). It was designed to depict a broadly relevant, non-diagnostic interpersonal difficulty that would be psychologically plausible and sufficiently self-relevant for a wide adult sample. Specifically, the scenario centered on a recurring interpersonal pattern involving feeling unseen, insufficiently appreciated, and strongly affected by criticism or lack of recognition from others. This scenario was selected because it was emotionally meaningful without requiring a specific diagnosis or prior treatment history, while also mapping directly onto the focal appraisal domains of the present study: perceived recognition, legitimacy, therapist authority, and autonomy-related resistance. This feature was important for the public mental health orientation of the study, because the aim was to examine early engagement processes among potential users of psychological care rather than only among patients already embedded in treatment.

Using a single clinical scenario while varying only the therapist’s response style allowed us to isolate the interpersonal meaning of the therapist’s intervention while holding the patient’s presenting problem constant. Because the study employed a between-subjects design, each participant was exposed to only one therapist response. This design allowed the two experimental conditions to be differentiated clearly without concern for carryover effects or direct within-person comparison. The vignette was presented in the second person to facilitate identification with the patient role.

The shared opening prompt presented to all participants was as follows:

*“Imagine that you are at the beginning of psychological treatment. During one of the first sessions, you tell the psychologist that in important relationships in your life, something similar keeps happening again and again: you feel that people do not truly see you, do not fully understand how deeply things affect you, and do not appreciate you as much as you would expect. You explain that when someone criticizes you, ignores you, or does not treat you in a way that feels appropriate to you, it affects you very strongly. Sometimes you react with anger, sometimes by withdrawing, and sometimes by becoming highly defensive. You add that you have already begun to notice that this is a recurring pattern, and that you would like to understand why this keeps happening and how it might be changed.”*

#### 2.4.2. Therapist-Response Conditions

Following the shared opening prompt, participants were randomly assigned to read one of two therapist responses.

In the validation-based condition, the therapist’s response was designed to convey empathic understanding, explicit acknowledgment of the legitimacy of the participant’s experience, and a collaborative, non-imposing invitation to explore the pattern together. The wording was intended to communicate understanding, respect, and psychological safety while preserving the participant’s sense of agency. The therapist’s response in this condition was as follows:

*“I can hear how painful it is for you to feel that people do not truly see you, do not grasp how deeply these things affect you, and do not give enough recognition to what you are experiencing. From what you are describing, it sounds as though your reactions are neither exaggerated nor arbitrary, but rather connected to deep experiences of hurt, disappointment, and perhaps also to a sense that there has not been enough room for what you have been feeling over time. In that sense, it is very understandable that when situations like these happen again, you respond intensely.*

*What feels most important to me right now is not to rush to judge you, not to decide too quickly what is wrong, and not to impose an explanation on you from the outside. First of all, I would want us to understand together how this feels from the inside for you: in what kinds of situations this gets activated, what exactly happens for you in those moments, what you feel, and what meaning it carries for you. If it feels right for you, we can gradually begin to explore how this pattern may have developed, what it may be trying to protect, and what new ways of coping might help you respond differently. But we will do this together, at a pace that feels safe for you, and in a way that allows you to feel genuinely understood and given real space.”*

In the challenge-based condition, the therapist’s response was designed to be more interpretive, authoritative, and change-oriented. The wording identified the problematic pattern more explicitly, placed stronger emphasis on the participant’s responsibility for change, and was intended to feel more imposing, more evaluatively framed, and less autonomy-supportive than the validation-based condition, while still remaining professionally plausible and clinically credible. The therapist’s response in this condition was as follows:

*“What you are describing sounds like a clear and deeply rooted pattern in which you react with excessive intensity to situations involving criticism, lack of attention, or lack of appreciation. It is important to say this plainly: the problem is not only in what other people do, but also in the way you interpret these situations and in how you respond to them. It seems that you tend to feel hurt very quickly, move into a defensive position, and then react in ways that intensify the difficulty rather than reduce it.*

*If you truly want something to change, you will need to start identifying this pattern earlier, stop automatically justifying it, and take clear responsibility for it. In therapy, we will need to work in a direct and focused way on how you interpret other people’s behavior, on your strong need for recognition and approval from them, and on your tendency to react out of hurt, anger, or defensiveness. This will require a willingness on your part to look at yourself honestly, even when that is uncomfortable. Without directly confronting this pattern and making a real effort to change it, it will be very difficult to achieve meaningful change.”*

The two therapist responses were matched as closely as possible in overall length, readability, professional tone, and apparent therapist competence. Thus, the primary difference between conditions was intended to be the interpersonal style and psychological meaning of the therapist’s intervention rather than its realism or clinical plausibility.

#### 2.4.3. Expert Validation of the Vignettes

Prior to the main study, the final vignette pair was reviewed by eight expert judges. Each judge read both vignettes, with order of presentation counterbalanced across judges, and rated the extent to which each therapist response was (a) validating, recognizing, and respectful toward the patient and (b) directive, authoritative, and challenging toward the patient. Ratings were made on a 5-point scale ranging from 1 (not at all) to 5 (very much). In addition, judges rated the realism, clarity, and naturalness of each vignette in order to assess the general plausibility and comparability of the two therapist responses.

Inter-rater agreement for the two core manipulation dimensions was assessed using intraclass correlation coefficients, ICC(3,1), because the same fixed panel of judges rated both vignettes. Inter-rater reliability was high for the validation-recognition-respect rating [ICC(3,1) = 0.97] and for the directive-authoritative-challenging rating [ICC(3,1) = 0.87].

As intended, the validation-based vignette was rated substantially higher on validation, recognition, and respect (*M* = 4.50, *SD* = 0.53) than the directive/challenging vignette (*M* = 1.75, *SD* = 0.71), *t*(7) = 16.80, *p* < 0.001, *CI*_95%_ [2.36, 3.14], Cohen’s *d_z_* = 5.94. Conversely, the directive/challenging vignette was rated substantially higher on directiveness, authoritativeness, and challenge (*M* = 4.25, *SD* = 0.89) than the validation-based vignette (*M* = 1.50, *SD* = 0.53), *t*(7) = 7.51, *p* < 0.001, *CI*_95%_ [1.88, 3.62], Cohen’s *d_z_* = 2.66.

Importantly, the two vignettes did not differ significantly in perceived realism, clarity, or naturalness. Ratings of realism were high for both the validation-based vignette (*M* = 4.63, *SD* = 0.52) and the directive/challenging vignette (*M* = 4.75, *SD* = 0.46), *t*(7) = −1.00, *p* = 0.351. Ratings of clarity were identical across conditions (both *Ms* = 4.75, *SDs* = 0.46), *t*(7) = 0.00, *p* = 1.000, and ratings of naturalness were likewise identical across conditions (both *Ms* = 4.75, *SDs* = 0.46), *t*(7) = 0.00, *p* = 1.000.

Taken together, these expert ratings support the content validity of the manipulation. The two vignettes differed strongly and in the intended direction on the focal interpersonal dimensions while remaining comparable in their overall realism, clarity, and naturalness.

### 2.5. Measures

Participants first completed a brief background questionnaire assessing demographic characteristics and psychotherapy-related background variables. Demographic variables included age, gender, education, employment status, relationship status, perceived socioeconomic status, and religiosity. Psychotherapy-related background variables included prior psychotherapy experience, current psychotherapy status, number of previous therapy episodes, cumulative duration of prior psychotherapy, current psychiatric medication use, past psychiatric medication use, and general familiarity with psychotherapy. In addition, participants completed one psychotherapy-evaluation item tailored to treatment history: participants with prior psychotherapy experience rated how helpful psychotherapy had been for them overall, whereas participants without prior psychotherapy experience rated the extent to which they believed psychotherapy can be helpful.

All questionnaire materials that were not originally available in Hebrew were translated from English into Hebrew and then back-translated into English by an independent bilingual translator. Discrepancies were resolved through discussion among the research team to ensure semantic equivalence.

#### 2.5.1. Narcissistic Admiration and Rivalry

Trait narcissism was assessed with the Narcissistic Admiration and Rivalry Questionnaire (NARQ) [22]. The measure includes 18 items assessing narcissistic admiration (9 items) and narcissistic rivalry (9 items). Participants responded on a 6-point scale ranging from 1 (not agree at all) to 6 (agree completely). Subscale scores were computed as item means, with higher scores indicating higher admiration or rivalry. Internal consistency was good for admiration (α = 0.83) and rivalry (α = 0.83).

#### 2.5.2. Perceived Recognition

Perceived recognition was assessed with an adapted 6-item scale from our prior work on recognition-related appraisals [29,30,31]. Items assessed the extent to which participants felt seen, understood, respected, and treated as legitimate by the therapist. Responses were made on a 7-point scale from 1 (strongly disagree) to 7 (strongly agree), and items were averaged. Internal consistency was excellent (α = 0.94).

#### 2.5.3. Perceived Freedom Threat

Perceived freedom threat was assessed with an adapted 4-item version of perceived threat-to-freedom items from the psychological reactance literature [13]. Items were reworded for the psychotherapy vignette context. Responses were made on a 5-point scale from 1 (strongly disagree) to 5 (strongly agree), and items were averaged. Internal consistency was acceptable (α = 0.78).

#### 2.5.4. State Reactance

State reactance was assessed with an adapted 10-item scale based on the Salzburger State Reactance Scale and related reactance items [13,32]. Items captured immediate anger, irritation, and resistant or oppositional cognitions in response to the therapist’s response. Responses were made on a 5-point scale from 1 (strongly disagree) to 5 (strongly agree), and items were averaged. Internal consistency was excellent (α = 0.96). For the primary analyses, perceived freedom threat and state reactance were standardized and averaged to form the autonomy-related resistance index. Supplementary analyses examined the two components separately.

#### 2.5.5. Anticipated Therapeutic Alliance

Anticipated therapeutic alliance was assessed with an adapted vignette-based version of the Working Alliance Inventory-Short Revised (WAI-SR) [33]. The 12 items were reworded so that participants rated the extent to which they expected they could develop a constructive working relationship with the therapist depicted in the vignette. Responses were made on a 5-point scale from 1 (never) to 5 (always), and items were averaged. Internal consistency was excellent (α = 0.93).

#### 2.5.6. Therapist Credibility and Expected Benefit

Therapist credibility and expected treatment benefit were assessed with an adapted version of the Credibility/Expectancy Questionnaire (CEQ) [34]. The adapted measure included six items referring to the therapist and treatment approach described in the vignette. Credibility and expected treatment benefit were scored separately after standardizing the relevant items, consistent with the original scoring approach. Higher scores indicated greater therapist credibility and greater expected treatment benefit. Internal consistency was good for credibility (α = 0.87) and expected treatment benefit (α = 0.89).

#### 2.5.7. Willingness to Continue with the Therapist

Willingness to continue with the therapist was assessed with a study-specific 3-item scale capturing immediate motivational engagement with the therapist depicted in the vignette. Responses were made on a 7-point scale from 1 (strongly disagree) to 7 (strongly agree), and items were averaged. Internal consistency was excellent (α = 0.96).

#### 2.5.8. Manipulation Checks

To assess whether the manipulation was perceived as intended, participants completed two manipulation-check items after the primary vignette-based questionnaires. One item assessed the extent to which the therapist’s response was perceived as validating, recognizing, and respectful, and the other assessed the extent to which it was perceived as directive, authoritative, and challenging. Responses were made on a 5-point scale from 1 (not at all) to 5 (very much). The two items were analyzed separately because they represented distinct aspects of the manipulation rather than a single latent construct.

### 2.6. Data Analytic Strategy

Primary analyses followed the preregistered analytic framework and were conducted after the application of the exclusion criteria. Descriptive statistics, internal consistency coefficients, and bivariate correlations were first examined for all major variables. The two manipulation-check items were tested using independent-samples *t* tests comparing the two vignette conditions. We also conducted additional confirmatory factor analyses to evaluate the discriminant validity of the Perceived Recognition scale relative to conceptually adjacent therapy-related evaluations: anticipated therapeutic alliance, therapist credibility, and expected treatment benefit.

The primary hypothesis tests used conditional process analyses conducted with Hayes’ PROCESS macro [35]. Therapist-response condition was effect-coded (validation-based response = +1; challenge-based response = −1). Separate models were estimated for each outcome variable with therapist response style as the predictor variable, narcissistic admiration and rivalry as the moderators, and perceived recognition and autonomy-related resistance as mediators.

Conditional indirect effects were estimated using bias-corrected bootstrap confidence intervals based on 10,000 resamples. For each model, we report the regression coefficients, bootstrapped indirect effects, and the index of moderated mediation, along with conditional indirect effects within each therapist-response condition. In supplementary analyses, perceived freedom threat and state reactance were also examined separately in order to evaluate the robustness and interpretability of the resistance pathway.

In supplementary analyses, prior psychotherapy experience, current psychotherapy status, psychiatric medication use, general familiarity with psychotherapy, age, and gender were examined as covariates or exploratory moderators. However, the primary analyses remained focused on therapist response style, narcissistic admiration and rivalry, perceived recognition, autonomy-related resistance, and therapy-relevant anticipated outcomes.

### 2.7. Ethical Considerations

The study was approved by the Institutional Review Board of Jerusalem Multidisciplinary College (Approval No. 737; 6 April 2026). All participants provided informed consent electronically before beginning the survey. Because the study involved reading a brief psychotherapy vignette and reflecting on possible emotional reactions, it was considered to involve minimal risk. Participants were informed that they could discontinue participation at any time without penalty and received a debriefing statement at the end of the study.

### 2.8. Data Availability

To enhance transparency and reproducibility, the preregistration and study materials are openly available on the Open Science Framework (OSF) at https://osf.io/asq42, accessed on 1 July 2026. The repository includes the preregistration and the anonymized SPSS (Version 28.01.1) dataset used for the analyses.

## 3. Results

### 3.1. Background and Sociodemographic Variables

Table 1 summarizes the demographic and therapy-related characteristics of the analytic sample, presented separately by participant gender and therapist-response condition. The table includes age, psychotherapy background, educational attainment, employment status, marital status, household income, and religiosity. These variables are reported to document the composition of the sample and to evaluate the comparability of the experimental groups.

Baseline comparability across therapist-response conditions was evaluated using independent-samples *t* tests for continuous variables and *χ*^2^ tests for categorical variables. The validation-based and challenge-based groups did not differ significantly on the sociodemographic or background variables examined (all ps > 0.05).

The absence of baseline differences reduces concern that the experimental effects reflected pre-existing group differences rather than the therapist-response manipulation. For this reason, these background variables were not retained as covariates in the primary analyses.

### 3.2. Manipulation Checks

Manipulation-check analyses indicated that the vignette manipulation was perceived in the intended direction. Participants in the validation-based condition rated the therapist’s response as significantly more validating, recognizing, and respectful than participants in the challenge-based condition, *M* = 3.96, *SD* = 1.01 vs. *M* = 3.59, *SD* = 1.03, *t*(970) = 5.67, *p* < 0.001, Cohen’s d = 0.36. Conversely, participants in the challenge-based condition rated the therapist’s response as significantly more directive, authoritative, and challenging than participants in the validation-based condition, *M* = 3.78, *SD* = 1.00 vs. *M* = 3.32, *SD* = 1.03, *t*(970) = 7.05, *p* < 0.001, Cohen’s d = 0.45. Thus, participants differentiated the two therapist-response conditions in the expected direction, although the effect sizes were modest. This is important for interpretation because the participant-level manipulation checks were considerably smaller than the expert-validation effects reported above. The manipulation therefore appears to have been successful but not strong at the level of participants’ subjective differentiation between conditions. This more modest differentiation should be considered when interpreting the magnitude of the experimental condition effects.

### 3.3. Univariate Analyses

Table 2A reports condition-specific descriptive statistics for the focal study variables, and Table 2B reports their zero-order correlations within each experimental condition. These results provide the descriptive basis for the hypothesis tests reported below.

Skewness and kurtosis values did not indicate severe distributional deviations, so the planned parametric analyses were retained. The correlation matrix showed the expected strong positive associations among the therapy-related outcomes and strong associations of perceived recognition and autonomy-related resistance with these outcomes in opposite directions. Narcissistic admiration showed small positive associations with several favorable therapy-related outcomes, whereas narcissistic rivalry showed its clearest association with greater autonomy-related resistance and, to a lesser extent, lower perceived recognition. Because the full correlation pattern is reported in Table 2, the text focuses only on these central descriptive trends.

As can be seen in Table 3, consistent with expectations, participants in the validation-based condition reported significantly greater perceived recognition and lower autonomy-related resistance than those in the challenge-based condition. Participants in the validation-based condition also reported greater willingness to continue therapy compared with those in the challenge-based condition. However, the conditions did not differ with respect to anticipated therapeutic alliance, therapist credibility, or expected treatment benefit.

### 3.4. Discriminant Validity of the Perceived Recognition Scale

Because perceived recognition served as a central mediator in the present model, we conducted additional analyses to evaluate whether it was empirically distinguishable from conceptually adjacent therapy-related evaluations. Specifically, we tested a confirmatory factor model in which the perceived recognition items, anticipated therapeutic alliance items, therapist credibility items, and expected treatment benefit items loaded on four separate latent factors. Therapist credibility was represented by CEQ1–CEQ3, and expected treatment benefit was represented by CEQ4–CEQ6, consistent with the scoring approach used for the adapted Credibility/Expectancy Questionnaire.

The four-factor model showed adequate fit to the data, χ^2^(246) = 1796.86, *p* < 0.001, CFI = 0.917, TLI = 0.907, RMSEA = 0.081, SRMR = 0.052. In contrast, a one-factor model in which all items loaded on a single general positive-evaluation factor fit the data substantially worse, χ^2^(252) = 5907.54, *p* < 0.001, CFI = 0.697, TLI = 0.668, RMSEA = 0.152, SRMR = 0.096, Δχ^2^(6) = 4110.68, *p* < 0.001.

We also compared the four-factor model with alternative models in which perceived recognition was combined with anticipated therapeutic alliance, therapist credibility, or expected treatment benefit. Each alternative model fit the data substantially worse than the four-factor model, indicating that perceived recognition was not redundant with these adjacent constructs. The latent correlation between perceived recognition and anticipated therapeutic alliance was 0.679; the latent correlation between perceived recognition and therapist credibility was 0.604, and the latent correlation between perceived recognition and expected treatment benefit was 0.500. Standardized factor loadings for the perceived recognition items ranged from 0.779 to 0.897, and the average variance extracted for perceived recognition was 0.742. Taken together, these findings suggest that perceived recognition is related to favorable therapy-related evaluations, as expected, but is empirically distinguishable from anticipated alliance, therapist credibility, and expected treatment benefit.

### 3.5. Perceived Recognition and Autonomy-Related Resistance

We began our analyses by conducting a parallel multiple mediation model for each outcome, with experimental condition as the predictor and perceived recognition and autonomy-related resistance as the potential mediators. As expected, experimental condition was significantly associated with both mediators. More specifically, it was positively associated with perceived recognition (*B* = 0.27, *CI*_95%_[0.19, 0.35], *SE* = 0.04, *t* = 6.60, *p* < 0.001) and negatively associated with autonomy-related resistance (*B* = −0.28, *CI*_95%_[−0.33, −0.22], *SE* = 0.03, *t* = −10.44, *p* < 0.001), such that participants in the validation-based condition reported greater perceived recognition and lower autonomy-related resistance than those in the challenge-based condition.

In turn, perceived recognition was positively associated with all outcomes: anticipated therapeutic alliance (*B* = 0.27, *CI*_95%_[0.25, 0.30], *SE* = 0.01, *t* = 19.25, *p* < 0.001), therapist credibility (*B* = 0.61, *CI*_95%_[0.53, 0.69], *SE* = 0.04, *t* = 15.31, *p* < 0.001), expected treatment benefit (*B* = 0.27, *CI*_95%_[0.23, 0.32], *SE* = 0.02, *t* = 12.57, *p* < 0.001), and willingness to continue therapy (*B* = 0.42, *CI*_95%_[0.38, 0.47], *SE* = 0.02, *t* = 18.06, *p* < 0.001). In contrast, autonomy-related resistance was negatively associated with these same outcomes: anticipated therapeutic alliance (*B* = −0.21, *CI*_95%_[−0.25, −0.17], *SE* = 0.02, *t* = −9.60, *p* < 0.001), therapist credibility (*B* = −0.55, *CI*_95%_[−0.67, −0.42], *SE* = 0.06, *t* = −8.81, *p* < 0.001), expected treatment benefit (*B* = −0.20, *CI*_95%_[−0.26, −0.13], *SE* = 0.03, *t* = −5.85, *p* < 0.001), and willingness to continue therapy (*B* = −0.32, *CI*_95%_[−0.39, −0.25], *SE* = 0.04, *t* = −8.81, *p* < 0.001).

Tests of indirect effects indicated that both mediators significantly accounted for the associations between the experimental condition and the outcomes. Perceived recognition mediated the effects of condition on anticipated therapeutic alliance (*B* = 0.07, *CI*_95%_[0.05, 0.10], *SE* = 0.01, *z* = 6.24, *p* < 0.001), therapist credibility (*B* = 0.17, *CI*_95%_[0.11, 0.22], *SE* = 0.03, *z* = 6.05, *p* < 0.001), expected treatment benefit (*B* = 0.07, *CI*_95%_[0.05, 0.10], *SE* = 0.01, *z* = 5.83, *p* < 0.001), and willingness to continue therapy (*B* = 0.11, *CI*_95%_[0.08, 0.15], *SE* = 0.02, *z* = 6.19, *p* < 0.001). Similarly, autonomy-related resistance mediated the effects of condition on anticipated therapeutic alliance (*B* = 0.06, *CI*_95%_[0.04, 0.08], *SE* = 0.01, *z* = 7.05, *p* < 0.001), therapist credibility (*B* = 0.15, *CI*_95%_[0.10, 0.20], *SE* = 0.02, *z* = 6.72, *p* < 0.001), expected treatment benefit (*B* = 0.05, *CI*_95%_[0.03, 0.08], *SE* = 0.01, *z* = 5.08, *p* < 0.001), and willingness to continue therapy (*B* = 0.09, *CI*_95%_[0.06, 0.12], *SE* = 0.01, *z* = 6.71, *p* < 0.001).

Taken together, these findings indicate that the validation-based condition was indirectly associated with more favorable therapy-related outcomes than the challenge-based condition through higher perceived recognition and lower autonomy-related resistance.

Following the parallel multiple mediation analyses, we conducted conditional process models to examine whether narcissistic admiration or narcissistic rivalry moderated the indirect effects of experimental condition on therapy-related outcomes via perceived recognition and autonomy-related resistance. Narcissistic admiration was positively associated with perceived recognition (*B* = 0.17, *CI*_95%_[0.07, 0.26], *SE* = 0.05, *t* = 3.47, *p* < 0.001), but it did not moderate the effect of experimental condition on perceived recognition (*B* = −0.02, *CI*_95%_[−0.12, 0.07], *SE* = 0.05, *t* = −0.50, *p* = 0.618). In addition, narcissistic admiration was not associated with autonomy-related resistance (*B* = 0.01, *CI*_95%_[−0.05, 0.07], *SE* = 0.03, *t* = 0.23, *p* = 0.822) and did not moderate the effect of experimental condition on autonomy-related resistance (*B* = −0.01, *CI*_95%_[−0.07, 0.05], *SE* = 0.03, *t* = −0.19, *p* = 0.848).

Narcissistic rivalry showed a different pattern of main effects. It was negatively associated with perceived recognition (*B* = −0.18, *CI*_95%_[−0.28, −0.07], *SE* = 0.05, *t* = −3.31, *p* = 0.001) and positively associated with autonomy-related resistance (*B* = 0.28, *CI*_95%_[0.22, 0.35], *SE* = 0.03, *t* = 8.44, *p* < 0.001). However, like narcissistic admiration, narcissistic rivalry did not moderate the effect of the experimental condition on perceived recognition (*B* = −0.01, *CI*_95%_[−0.11, 0.10], *SE* = 0.05, *t* = −0.16, *p* = 0.872) or autonomy-related resistance (*B* = −0.01, *CI*_95%_[−0.07, 0.06], *SE* = 0.03, *t* = −0.17, *p* = 0.863).

The results of the conditional process analyses indicate that although narcissistic admiration and rivalry exhibited distinct associations with the mediators, neither aspect of narcissism moderated the effects of experimental condition on perceived recognition or autonomy-related resistance.

Table 4, Table 5, Table 6 and Table 7 report the conditional process models for the four outcomes. The mediator models are identical across analyses and are reproduced in each table for completeness, whereas the outcome model changes according to the dependent variable. To reduce redundancy, the text below summarizes the hypothesis-relevant findings: direct condition effects, mediator-outcome paths, moderation tests, bootstrapped indirect effects, and indices of moderated mediation.

### 3.6. Anticipated Therapeutic Alliance

The results of the conditional process analysis predicting anticipated therapeutic alliance are presented in Table 4. After accounting for the mediators and moderators, experimental condition showed a negative residual direct association with anticipated therapeutic alliance (*B* = −0.19, *CI*_95%_[−0.34, −0.04], *SE* = 0.08, *t* = −2.50, *p* = 0.013). This indicates that, after perceived recognition and autonomy-related resistance were held constant, the residual association favored the challenge-based condition. This pattern should be interpreted in the context of the significant positive indirect effects reported below.

**Table 4:** Results of the conditional process analysis for anticipated therapeutic alliance.

	Outcome								
	M_1_: Perceived Recognition			M_2_: Autonomy-Related Resistance			Y: Anticipated Therapeutic Alliance		
Predictor	*B*	*SE*	*p*	*B*	*SE*	*p*	*B*	*SE*	*p*
X: Experimental Condition	0.38	0.20	0.062	−0.25	0.13	0.048	−0.19	0.08	0.013
W_1_: Narcissistic Admiration (ADM)	0.17	0.05	<0.001	0.01	0.03	0.822	0.07	0.02	<0.001
W_2_: Narcissistic Rivalry (RIV)	−0.18	0.05	0.001	0.28	0.03	<0.001	−0.01	0.02	0.670
M_1_: Perceived Recognition	–	–	–	–	–	–	0.27	0.01	<0.001
M_2_: Autonomy-Related Resistance	–	–	–	–	–	–	−0.22	0.02	<0.001
X × W_1_: Condition × ADM	−0.02	0.05	0.618	−0.01	0.03	0.848	0.01	0.02	0.701
X × W_2_: Condition × RIV	−0.01	0.05	0.872	−0.01	0.03	0.863	0.03	0.02	0.085
Constant	4.79	0.20	<0.001	−0.64	0.13	<0.001	1.96	0.10	<0.001
	*R*^2^ = 0.06			*R*^2^ = 0.16			*R*^2^ = 0.49		
	*F* = 13.18, *p* < 0.001			*F* = 37.78, *p* < 0.001			*F* = 130.20, *p* < 0.001		
Conditional Indirect Association of Experimental Condition with Anticipated Therapeutic Alliance through Perceived Recognition									
Condition	*Coeff.*		*Boot SE*		*Boot LCI*		*Boot UCI*		
Low Narcissistic Admiration	0.08		0.02		0.05		0.11		
High Narcissistic Admiration	0.07		0.02		0.03		0.10		
Low Narcissistic Rivalry	0.07		0.02		0.04		0.11		
High Narcissistic Rivalry	0.07		0.02		0.04		0.10		
Conditional Indirect Association of Experimental Condition with Anticipated Therapeutic Alliance through Autonomy-Related Resistance									
Condition	*Coeff.*		*Boot SE*		*Boot LCI*		*Boot UCI*		
Low Narcissistic Admiration	0.06		0.01		0.04		0.08		
High Narcissistic Admiration	0.06		0.01		0.04		0.09		
Low Narcissistic Rivalry	0.06		0.01		0.04		0.08		
High Narcissistic Rivalry	0.06		0.01		0.04		0.09		

Narcissistic admiration was positively associated with anticipated therapeutic alliance (*B* = 0.07, *CI*_95%_[0.03, 0.11], *SE* = 0.02, *t* = 3.77, *p* < 0.001), whereas narcissistic rivalry was not (*B* = −0.01, *CI*_95%_[−0.05, 0.03], *SE* = 0.02, *t* = −0.43, *p* = 0.670). Neither narcissistic admiration (*B* = 0.01, *CI*_95%_[−0.03, 0.04], *SE* = 0.02, *t* = 0.38, *p* = 0.701) nor narcissistic rivalry (*B* = 0.03, *CI*_95%_[0.00, 0.07], *SE* = 0.02, *t* = 1.73, *p* = 0.085) moderated the effect of the experimental condition on anticipated therapeutic alliance.

Tests of moderated mediation indicated that neither narcissistic admiration nor narcissistic rivalry moderated the indirect effects of experimental condition on anticipated therapeutic alliance. Specifically, narcissistic admiration did not moderate the indirect effects via perceived recognition (*B* = −0.01, *CI*_95%_[−0.03, 0.02], *SE* = 0.01) or autonomy-related resistance (*B* = 0.00, *CI*_95%_[−0.01, 0.01], *SE* = 0.01). Similarly, narcissistic rivalry did not moderate the indirect effect that experimental condition had with anticipated therapeutic alliance through perceived recognition (*B* = 0.00, *CI*_95%_[−0.03, 0.02], *SE* = 0.01) or autonomy-related resistance (*B* = 0.00, *CI*_95%_[−0.01, 0.02], *SE* = 0.01). These results provide no evidence that narcissistic admiration or narcissistic rivalry moderates either the direct or indirect effects of experimental condition on anticipated therapeutic alliance.

### 3.7. Therapist Credibility

The results of the conditional process analysis predicting therapist credibility are presented in Table 5. After accounting for the mediators and moderators, experimental condition showed a negative residual direct association with therapist credibility (*B* = −0.49, *CI*_95%_[−0.92, −0.06], *SE* = 0.22, *t* = −2.25, *p* = 0.025). This indicates that, after perceived recognition and autonomy-related resistance were held constant, the residual association favored the challenge-based condition. This pattern should be interpreted in the context of the significant positive indirect effects reported below.

**Table 5:** Results of the conditional process analysis for therapist credibility.

	Outcome								
	M_1_: Perceived Recognition			M_2_: Autonomy-Related Resistance			Y: Therapist Credibility		
Predictor	*B*	*SE*	*p*	*B*	*SE*	*p*	*B*	*SE*	*p*
X: Experimental Condition	0.38	0.20	0.062	−0.25	0.13	0.048	−0.49	0.22	0.025
W_1_: Narcissistic Admiration (ADM)	0.17	0.05	<0.001	0.01	0.03	0.822	0.15	0.05	0.004
W_2_: Narcissistic Rivalry (RIV)	−0.18	0.05	0.001	0.28	0.03	<0.001	0.07	0.06	0.215
M_1_: Perceived Recognition	–	–	–	–	–	–	0.59	0.04	<0.001
M_2_: Autonomy-Related Resistance	–	–	–	–	–	–	−0.58	0.06	<0.001
X × W_1_: Condition × ADM	−0.02	0.05	0.618	−0.01	0.03	0.848	0.00	0.05	0.978
X × W_2_: Condition × RIV	−0.01	0.05	0.872	−0.01	0.03	0.863	0.09	0.06	0.127
Constant	4.79	0.20	<0.001	−0.64	0.13	<0.001	2.29	0.28	<0.001
	*R*^2^ = 0.06			*R*^2^ = 0.16			*R*^2^ = 0.39		
	*F* = 13.18, *p* < 0.001			*F* = 37.78, *p* < 0.001			*F* = 89.04, *p* < 0.001		
Conditional Indirect Association of Experimental Condition with Therapist Credibility through Perceived Recognition									
Condition	*Coeff.*		*Boot SE*		*Boot LCI*		*Boot UCI*		
Low Narcissistic Admiration	0.17		0.04		0.11		0.25		
High Narcissistic Admiration	0.15		0.04		0.08		0.23		
Low Narcissistic Rivalry	0.17		0.04		0.09		0.24		
High Narcissistic Rivalry	0.16		0.03		0.09		0.23		
Conditional Indirect Association of Experimental Condition with Therapist Credibility through Autonomy-Related Resistance									
Condition	*Coeff.*		*Boot SE*		*Boot LCI*		*Boot UCI*		
Low Narcissistic Admiration	0.16		0.03		0.11		0.23		
High Narcissistic Admiration	0.17		0.03		0.11		0.24		
Low Narcissistic Rivalry	0.16		0.03		0.10		0.23		
High Narcissistic Rivalry	0.17		0.03		0.11		0.23		

Narcissistic admiration was positively associated with therapist credibility (*B* = 0.15, *CI*_95%_[0.05, 0.26], *SE* = 0.05, *t* = 2.93, *p* = 0.004), whereas narcissistic rivalry was not (*B* = 0.07, *CI*_95%_[−0.04, 0.19], *SE* = 0.06, *t* = 1.24, *p* = 0.215). Neither narcissistic admiration (*B* = 0.00, *CI*_95%_[−0.10, 0.10], *SE* = 0.05, *t* = −0.03, *p* = 0.979) nor narcissistic rivalry (*B* = 0.09, *CI*_95%_[−0.02, 0.20], *SE* = 0.06, *t* = 1.53, *p* = 0.127) moderated the effect of experimental condition on therapist credibility.

Tests of moderated mediation indicated that neither narcissistic admiration nor narcissistic rivalry moderated the indirect effects of the experimental condition on therapist credibility. Specifically, narcissistic admiration did not moderate the indirect effects via perceived recognition (*B* = −0.01, *CI*_95%_[−0.07, 0.05], *SE* = 0.03) or autonomy-related resistance (*B* = 0.00, *CI*_95%_[−0.03, 0.04], *SE* = 0.02). Similarly, narcissistic rivalry did not moderate the indirect effect that the experimental condition had on therapist credibility through perceived recognition (*B* = −0.01, *CI*_95%_[−0.07, 0.06], *SE* = 0.03) or autonomy-related resistance (*B* = 0.00, *CI*_95%_[−0.04, 0.04], *SE* = 0.02). These results provide no evidence that narcissistic admiration or rivalry moderates either the direct or indirect effects of the experimental condition on therapist credibility.

### 3.8. Expected Treatment Benefit

The results of the conditional process analysis predicting expected treatment benefit are presented in Table 6. After accounting for the mediators and moderators, experimental condition was not associated with expected treatment benefit (*B* = −0.17, *CI*_95%_[−0.40, 0.06], *SE* = 0.12, *t* = −1.47, *p* = 0.143), such that participants in the validation-based condition did not differ from those in the challenge-based condition with respect to expected treatment benefit.

**Table 6:** Results of the conditional process analysis for expected treatment benefit.

	Outcome								
	M_1_: Perceived Recognition			M_2_: Autonomy-Related Resistance			Y: Expected Treatment Benefit		
Predictor	*B*	*SE*	*p*	*B*	*SE*	*p*	*B*	*SE*	*p*
X: Experimental Condition	0.38	0.20	0.062	−0.25	0.13	0.048	−0.17	0.12	0.143
W_1_: Narcissistic Admiration (ADM)	0.17	0.05	<0.001	0.01	0.03	0.822	0.12	0.03	<0.001
W_2_: Narcissistic Rivalry (RIV)	−0.18	0.05	0.001	0.28	0.03	<0.001	−0.03	0.03	0.315
M_1_: Perceived Recognition	–	–	–	–	–	–	0.26	0.02	<0.001
M_2_: Autonomy-Related Resistance	–	–	–	–	–	–	−0.20	0.03	<0.001
X × W_1_: Condition × ADM	−0.02	0.05	0.618	−0.01	0.03	0.848	−0.01	0.03	0.829
X × W_2_: Condition × RIV	−0.01	0.05	0.872	−0.01	0.03	0.863	0.04	0.03	0.238
Constant	4.79	0.20	<0.001	−0.64	0.13	<0.001	−1.66	0.15	<0.001
	*R*^2^ = 0.06			*R*^2^ = 0.16			*R*^2^ = 0.29		
	*F* = 13.18, *p* < 0.001			*F* = 37.78, *p* < 0.001			*F* = 55.17, *p* < 0.001		
Conditional Indirect Association of Experimental Condition with Expected Treatment Benefit through Perceived Recognition									
Condition	*Coeff.*		*Boot SE*		*Boot LCI*		*Boot UCI*		
Low Narcissistic Admiration	0.08		0.02		0.05		0.11		
High Narcissistic Admiration	0.07		0.02		0.03		0.10		
Low Narcissistic Rivalry	0.07		0.02		0.04		0.11		
High Narcissistic Rivalry	0.07		0.02		0.04		0.10		
Conditional Indirect Association of Experimental Condition with Expected Treatment Benefit through Autonomy-Related Resistance									
Condition	*Coeff.*		*Boot SE*		*Boot LCI*		*Boot UCI*		
Low Narcissistic Admiration	0.06		0.01		0.03		0.08		
High Narcissistic Admiration	0.06		0.01		0.03		0.09		
Low Narcissistic Rivalry	0.06		0.01		0.03		0.09		
High Narcissistic Rivalry	0.06		0.01		0.03		0.09		

Narcissistic admiration was positively associated with expected treatment benefit (*B* = 0.12, *CI*_95%_[0.07, 0.18], *SE* = 0.03, *t* = 4.33, *p* < 0.001), whereas narcissistic rivalry was not (*B* = −0.03, *CI*_95%_[−0.09, 0.03], *SE* = 0.03, *t* = −1.01, *p* = 0.315). Neither narcissistic admiration (*B* = −0.01, *CI*_95%_[−0.06, 0.05], *SE* = 0.03, *t* = −0.22, *p* = 0.829) nor narcissistic rivalry (*B* = 0.04, *CI*_95%_[−0.02, 0.10], *SE* = 0.03, *t* = 1.18, *p* = 0.238) moderated the effect of experimental condition on expected treatment benefit.

Tests of moderated mediation indicated that neither narcissistic admiration nor narcissistic rivalry moderated the indirect effects of experimental condition on expected treatment benefit. Specifically, narcissistic admiration did not moderate the indirect effects via perceived recognition (*B* = −0.01, *CI*_95%_[−0.03, 0.02], *SE* = 0.01) or autonomy-related resistance (*B* = 0.00, *CI*_95%_[−0.01, 0.01], *SE* = 0.01). Similarly, narcissistic rivalry did not moderate the indirect effect that experimental condition had with expected treatment benefit through perceived recognition (*B* = 0.00, *CI*_95%_[−0.03, 0.02], *SE* = 0.01) or autonomy-related resistance (*B* = 0.00, *CI*_95%_[−0.01, 0.01], *SE* = 0.01). These results provide no evidence that narcissistic admiration or rivalry moderates either the direct or indirect effects of experimental condition on expected treatment benefit.

### 3.9. Willingness to Continue Therapy

The results of the conditional process analysis predicting willingness to continue therapy are presented in Table 7. After accounting for the mediators and moderators, the experimental condition showed no residual direct association with willingness to continue therapy (*B* = 0.08, *CI*_95%_[−0.17, 0.32], *SE* = 0.13, *t* = 0.60, *p* = 0.550). This indicates that the simple condition difference in willingness to continue therapy was accounted for by the appraisal pathways, particularly perceived recognition and autonomy-related resistance.

**Table 7:** Results of the conditional process analysis for willingness to continue therapy.

	Outcome								
	M_1_: Perceived Recognition			M_2_: Autonomy-Related Resistance			Y: Willingness to Continue Therapy		
Predictor	*B*	*SE*	*p*	*B*	*SE*	*p*	*B*	*SE*	*p*
X: Experimental Condition	0.38	0.20	0.062	−0.25	0.13	0.048	0.08	0.13	0.550
W_1_: Narcissistic Admiration (ADM)	0.17	0.05	<0.001	0.01	0.03	0.822	0.06	0.03	0.050
W_2_: Narcissistic Rivalry (RIV)	−0.18	0.05	0.001	0.28	0.03	<0.001	0.09	0.03	0.007
M_1_: Perceived Recognition	–	–	–	–	–	–	0.41	0.02	<0.001
M_2_: Autonomy-Related Resistance	–	–	–	–	–	–	−0.35	0.04	<0.001
X × W_1_: Condition × ADM	−0.02	0.05	0.618	−0.01	0.03	0.848	−0.04	0.03	0.243
X × W_2_: Condition × RIV	−0.01	0.05	0.872	−0.01	0.03	0.863	−0.02	0.03	0.493
Constant	4.79	0.20	<0.001	−0.64	0.13	<0.001	−1.66	0.15	<0.001
	*R*^2^ = 0.06			*R*^2^ = 0.16			*R*^2^ = 0.45		
	*F* = 13.18, *p* < 0.001			*F* = 37.78, *p* < 0.001			*F* = 114.41, *p* < 0.001		
Conditional Indirect Association of Experimental Condition with Willingness to Continue Therapy through Perceived Recognition									
Condition	*Coeff.*		*Boot SE*		*Boot LCI*		*Boot UCI*		
Low Narcissistic Admiration	0.12		0.02		0.07		0.17		
High Narcissistic Admiration	0.10		0.03		0.05		0.16		
Low Narcissistic Rivalry	0.11		0.03		0.06		0.17		
High Narcissistic Rivalry	0.11		0.02		0.07		0.16		
Conditional Indirect Association of Experimental Condition with Willingness to Continue Therapy through Autonomy-Related Resistance									
Condition	*Coeff.*		*Boot SE*		*Boot LCI*		*Boot UCI*		
Low Narcissistic Admiration	0.10		0.02		0.07		0.13		
High Narcissistic Admiration	0.10		0.02		0.07		0.14		
Low Narcissistic Rivalry	0.10		0.02		0.06		0.14		
High Narcissistic Rivalry	0.10		0.02		0.07		0.14		

Narcissistic admiration (*B* = 0.06, *CI*_95%_[0.00, 0.12], *SE* = 0.03, *t* = 1.96, *p* = 0.050) and narcissistic rivalry (*B* = 0.09, *CI*_95%_[0.03, 0.16], *SE* = 0.03, *t* = 2.72, *p* = 0.007) were both positively associated with willingness to continue therapy. Neither narcissistic admiration (*B* = −0.04, *CI*_95%_[−0.10, 0.02], *SE* = 0.03, *t* = −1.17, *p* = 0.243) nor narcissistic rivalry (*B* = −0.02, *CI*_95%_[−0.09, 0.04], *SE* = 0.03, *t* = −0.69, *p* = 0.493) moderated the effect of experimental condition on willingness to continue therapy.

Tests of moderated mediation indicated that neither narcissistic admiration nor narcissistic rivalry moderated the indirect effects of the experimental condition on willingness to continue therapy. Specifically, narcissistic admiration did not moderate the indirect effects via perceived recognition (*B* = −0.01, *CI*_95%_[−0.05, 0.03], *SE* = 0.02) or autonomy-related resistance (*B* = 0.00, *CI*_95%_[−0.02, 0.02], *SE* = 0.01). Similarly, narcissistic rivalry did not moderate the indirect effect that the experimental condition had on willingness to continue therapy through perceived recognition (*B* = 0.00, *CI*_95%_[−0.05, 0.04], *SE* = 0.02) or autonomy-related resistance (*B* = 0.00, *CI*_95%_[−0.02, 0.03], *SE* = 0.01). These results provide no evidence that narcissistic admiration or rivalry moderates either the direct or indirect effects of the experimental condition on willingness to continue therapy.

## 4. Discussion

The present study examined early psychotherapy engagement as a public mental health process. Specifically, we tested how potential users of psychological care appraise an initial therapist response and whether these appraisals shape anticipated alliance, therapist credibility, expected treatment benefit, and willingness to continue care. Drawing on a recognition–resistance framework, we compared a validation-based, recognition-supportive, autonomy-supportive therapist response with a more directive and challenging therapist response while holding the patient’s presenting concern constant. The study focused on two proximal appraisal pathways, perceived recognition and autonomy-related resistance, that may help explain how early therapist communication influences anticipated engagement with psychological care.

The findings provide strong support for the central appraisal-process component of the model, although not for the moderated mediation hypotheses. The manipulation operated as intended: participants in the validation-based condition perceived the response as more validating, recognizing, and respectful, whereas those in the directive and challenging condition perceived it as more directive, authoritative, and challenging. The validation-based response also increased perceived recognition and reduced autonomy-related resistance, indicating that relatively brief therapist responses can carry distinct interpersonal meanings even when embedded within the same clinical scenario and presented in a standardized vignette format.

The simple condition effects offered partial support for H1. As expected, participants in the validation-based condition reported greater perceived recognition, lower autonomy-related resistance, and a stronger willingness to continue working with the therapist than participants who received the directive and challenging response. However, the two conditions did not differ significantly in anticipated therapeutic alliance, therapist credibility, or expected treatment benefit. These findings suggest that the validation-based response did not uniformly enhance all therapy-related evaluations at the level of simple mean differences.

This pattern is broadly consistent with psychotherapy research showing that relational qualities such as alliance, empathy, positive regard, and therapist responsiveness play important roles in treatment process and outcome [1,2,3,4]. At the same time, it aligns with evidence indicating that therapist challenge and other directive interventions can also be clinically valuable, with their effects depending in part on how they are delivered and how they are experienced by clients [6,7]. Similarly, research on client preferences and psychological reactance suggests that directive or challenging therapist behaviors may be perceived as constructive and helpful by some individuals, but as controlling or autonomy-threatening by others [8,9,10,11,13,14,15].

Notably, the strongest and most consistent effects of therapist response style emerged for the proximal appraisal variables rather than the broader therapy-related outcomes. This pattern is consistent with the possibility that therapist communication initially influences how clients interpret and make sense of the therapeutic encounter—particularly whether they feel recognized and whether they experience resistance related to autonomy concerns—and that these appraisals subsequently shape more global evaluations of the therapist and the potential therapeutic relationship.

The findings for H2 were highly consistent with the proposed recognition–resistance framework. Perceived recognition was positively associated with anticipated therapeutic alliance, therapist credibility, expected treatment benefit, and willingness to continue with the therapist, whereas autonomy-related resistance was negatively associated with each of these outcomes. This pattern aligns with the broader psychotherapy literature showing that relational qualities such as therapeutic alliance, therapist empathy, positive regard, and responsiveness are central to psychotherapy process and outcome [1,2,3,4]. It is also consistent with research on client preferences, autonomous motivation, and psychological reactance, which suggests that engagement is strengthened when clients experience therapist behavior as responsive and autonomy-supportive, but weakened when interpersonal messages are experienced as controlling or freedom-threatening [8,9,10,11,12,13,14,15,27,28]. The present study extends this literature by showing that even a brief therapist response can evoke clinically meaningful appraisals concerning recognition, autonomy, and anticipated engagement.

The indirect effects provided further support for H3. The validation-based condition was indirectly associated with more favorable outcomes, including anticipated alliance, therapist credibility, expected treatment benefit, and willingness to continue therapy, through higher perceived recognition and lower autonomy-related resistance. This mediated pattern is consistent with evidence that therapist responsiveness, empathy, positive regard, verbal interventions, and challenge are consequential partly because of how patients interpret the interpersonal meaning of the therapist’s behavior [1,2,3,4,5,6,7]. It also aligns with research on client preferences, psychological reactance, and autonomous motivation, which suggests that engagement is more likely when therapist communication is experienced as responsive and autonomy-supportive rather than controlling or freedom-threatening [8,9,10,11,12,13,14,15,27,28]. The interpersonal meaning participants assigned to the therapist response was therefore more informative than therapist response style alone: the central issue was not simply whether the therapist validated or challenged, but whether the response was experienced as recognizing and legitimizing or as pressuring and autonomy-threatening.

The residual direct effects of condition should also be interpreted cautiously. In some models, most clearly for anticipated alliance and therapist credibility, the residual direct association favored the challenge-based condition after perceived recognition and autonomy-related resistance were held constant. This pattern is consistent with a competitive mediation or suppression structure [36], but it should not be interpreted as evidence that directive challenge is generally superior to validation-based responding. Rather, it suggests that once the central appraisal pathways of recognition and resistance are statistically equated, some remaining features of the challenge-based response, such as perceived seriousness, focus, directness, or professional authority, may account for residual variance in certain anticipated evaluations. Because these features were not directly measured, this interpretation remains tentative. The most robust interpretable finding is therefore the positive indirect pathway: the validation-based response promoted more favorable anticipated outcomes insofar as it increased perceived recognition and reduced autonomy-related resistance. Future studies should measure perceived therapist seriousness, structure, expertise, and authority directly in order to clarify whether these residual direct effects reflect meaningful features of challenge-based communication or model-dependent suppression.

This dual-pathway pattern is theoretically important because it supports the distinction between recognition-related and resistance-related appraisals. Perceived recognition and autonomy-related resistance were related but not reducible to one another. Feeling recognized is not merely the absence of threat, and feeling threatened is not simply the absence of recognition. This distinction fits with psychological reactance theory, which conceptualizes resistance as a motivational response to threatened freedom rather than merely as negative evaluation [13,14,15]. It also fits with psychotherapy research emphasizing client preferences, therapist directiveness, and treatment responsiveness [8,9,10,11]. A therapist may be experienced as challenging yet respectful, or as warm yet subtly imposing. The present findings suggest that these distinctions matter for anticipated therapeutic engagement.

The present findings should also be understood as part of a broader program of research on recognition and resistance as dual appraisal pathways in narcissistic self-regulation. Across our recent studies, perceived recognition has emerged as a central mechanism through which socially meaningful cues shape downstream evaluations, affective responses, and behavioral intentions. In older adults’ evaluations of empathic versus cold socially assistive robots, perceived recognition helped explain how narcissistic admiration and rivalry were linked to evaluations of empathic technological agents while also showing that empathic responsiveness was not uniformly beneficial for individuals higher in rivalry-related threat sensitivity [29]. In the context of self-relevant advertising, responses were similarly organized around partially distinct pathways of perceived recognition and autonomy-related resistance, with admiration showing stronger links to recognition-based responding and rivalry showing a more conflicted pattern involving both recognition and resistance [30]. Most directly, in a playground norm-framing study, recognition-based versus status-challenging framing strongly influenced perceived recognition and perceived freedom threat, which in turn predicted reactance-related, affective, evaluative, and behavioral responses, whereas narcissistic admiration and rivalry did not moderate the indirect effects [31].

The present study should therefore be understood as an independent extension of the recognition–resistance framework rather than as a replication of the playground norm-framing study. Although both studies examine recognition-related and autonomy-related appraisals, they differ in their substantive setting, target population, experimental stimulus, and outcome domain. The playground study examined public parenting encounters among young parents and focused on norm-framing, freedom threat, affective responses, and behavioral intentions in a community context [31]. In contrast, the present study examines early psychotherapeutic encounters among a broad adult sample and focuses on therapist response style, anticipated therapeutic alliance, therapist credibility, expected treatment benefit, and willingness to continue psychological care. These differences are important because psychotherapy involves asymmetrical expertise, emotional disclosure, dependence, expectations of self-examination, and decisions about continued engagement with mental health services. Thus, the present findings do not merely reproduce the earlier paradigm in a new setting. They suggest that recognition and autonomy-related resistance are relevant to early psychotherapy engagement, a public mental health process with implications for treatment uptake, early retention, and access to effective psychological care. The psychotherapy context adds a distinctive layer to this broader pattern. Unlike advertising, human–robot interaction, or a brief public parenting encounter, psychotherapy is a structured helping relationship marked by asymmetrical expertise, emotional disclosure, dependence, and the expectation of self-examination and change. Therapist challenge may therefore carry a more complex meaning here than in everyday social interaction: a directive and challenging response may evoke resistance when it feels imposing or insufficiently recognizing, but it may also communicate seriousness, expertise, focus, and a commitment to change. This dual potential may help explain why the validation-based condition clearly improved recognition and reduced resistance but did not produce uniformly stronger direct effects on all downstream outcomes.

The findings concerning narcissistic admiration and rivalry were also informative. Narcissistic admiration was positively associated with perceived recognition and showed positive associations with the therapy-related outcomes. This pattern is consistent with the Narcissistic Admiration and Rivalry Concept [22], which characterizes admiration as an agentic and self-enhancing orientation organized around visibility, affirmation, and social approval. Individuals higher in admiration may be especially receptive to interpersonal cues that allow them to feel seen, respected, and taken seriously. In the present context, this was reflected not in stronger sensitivity to the experimental manipulation, but in a more favorable general appraisal and outcome profile.

Narcissistic rivalry showed a different and more complex pattern. Consistent with theory, rivalry was associated with lower perceived recognition and greater autonomy-related resistance. This fits the view of rivalry as a defensive, antagonistic, status-protective orientation that is especially sensitive to perceived threat, criticism, or diminished agency [22,23,24,25]. It is also consistent with evidence that rivalry is more strongly associated with emotion regulation difficulties and defensive responding than admiration [26]. In this sense, the findings support the idea that rivalry may complicate early therapeutic engagement by increasing the likelihood that therapist behavior will be appraised through a lens of insufficient recognition and autonomy threat.

However, narcissistic rivalry also produced an unexpected and theoretically open finding. In the conditional process model predicting willingness to continue therapy, rivalry was positively associated with willingness to continue after perceived recognition and autonomy-related resistance were included in the model. This residual association should not be interpreted as evidence that narcissistic rivalry generally facilitates therapeutic engagement. The broader pattern showed that rivalry was associated with lower perceived recognition and greater autonomy-related resistance, both of which were associated with lower willingness to continue. Thus, the positive residual coefficient is directionally different from the indirect pattern and may reflect suppression, omitted residual mechanisms, or the low-stakes nature of the analogue vignette context.

One tentative possibility is that, after the immediate experience of insufficient recognition and autonomy-related resistance is statistically controlled, rivalry may retain a small residual association with perceiving therapy as serious, consequential, or worth continuing. However, the present study did not directly measure perceived seriousness, therapist authority, self-relevant challenge, mastery motivation, or perceived opportunity to confront interpersonal difficulties. This interpretation therefore remains speculative. It is also possible that this residual effect is specific to an online vignette completed privately by non-patients, where there is no actual therapist, no real risk of negative evaluation, and no genuine dependence on treatment. In real clinical settings, where vulnerability, exposure, and dependence are more salient, rivalry-related defensiveness may operate differently and may predict rupture, disengagement, or dropout rather than willingness to continue [16,17,18,23]. The residual positive association between rivalry and willingness to continue should therefore be treated as anomalous and hypothesis-generating rather than as a stable or generalizable feature of narcissistic rivalry in psychotherapy.

The absence of moderation by narcissistic admiration and rivalry requires a more cautious interpretation than originally anticipated. H4 and H5 predicted that admiration and rivalry would moderate the associations between therapist response style and the appraisal variables, and that they would also moderate the indirect effects of therapist response style on the outcomes. These hypotheses were not supported. The therapist-response manipulation affected perceived recognition and autonomy-related resistance in broadly similar ways across levels of admiration and rivalry. Thus, narcissistic traits did not function as differential susceptibility variables in the predicted manner, and the moderated-mediation component of the model was not supported.

The repeated absence of moderation by narcissistic admiration and rivalry also has implications beyond the present study. Across our related recognition–resistance studies, the appraisal-pathway component has been more consistently supported than the differential-susceptibility component involving admiration and rivalry as moderators. This cumulative pattern should not be treated as evidence for a successful refinement of the moderation model. Rather, it indicates that the moderated-mediation component of the framework remains unsupported in these analogue paradigms and should be regarded as provisional pending independent replication, stronger situational activation of narcissistic self-regulatory dynamics, and tests in more ecologically valid interpersonal and clinical contexts. Accordingly, the present findings support a more limited conclusion: therapist response style may shape recognition-related and autonomy-related appraisals, and narcissistic admiration and rivalry may be associated with general appraisal tendencies, but the data do not show that admiration and rivalry reliably identify for whom therapist response style has stronger or weaker effects.

At the same time, cross-study convergence should be interpreted cautiously. The related studies were conducted by the same research group, used conceptually similar vignette-based paradigms, and relied on related perceived-recognition measures. Thus, although the present discriminant-validity analyses suggest that perceived recognition is not merely a redundant proxy for anticipated alliance, therapist credibility, or expected treatment benefit in this dataset, independent validation and replication are needed before stronger claims are made about the generality of the recognition–resistance framework across contexts.

This finding limits the theoretical contribution of narcissistic admiration and rivalry in the present study. Rather than showing that admiration and rivalry condition the impact of therapist response style, the data suggest that these traits operated primarily as additive appraisal and outcome tendencies. Admiration was associated with more favorable recognition-related and therapy-related responses, whereas rivalry was associated with less recognition and more resistance, but also with the unexpected residual willingness-to-continue effect discussed above. This interpretation should be treated as a post hoc account of the observed pattern rather than as confirmation of the preregistered moderation hypotheses. Importantly, this more cautious interpretation does not change the distinctive contribution of the present study, which concerns early psychotherapeutic encounters rather than everyday community encounters, consumer communication, or human-technology interaction. The present findings therefore support the recognition–resistance component of the model more strongly than the trait-based differential-susceptibility component. Narcissistic admiration and rivalry may still be relevant to early psychotherapy engagement, but in this analogue context their role appears closer to trait covariates or general appraisal orientations than to moderators of experimentally manipulated therapist response style.

### 4.1. Implications for Behavioral and Mental Health Promotion

The present findings have implications not only for psychotherapy-process research but also for public mental health, behavioral health promotion, and service engagement. Early engagement in psychotherapy is an issue relevant to public health because individuals’ first appraisals of a therapist may shape treatment expectations, willingness to continue, and openness to receiving psychological support. From this perspective, the therapeutic encounter is not only a clinical event but also a behavioral and social context in which recognition, autonomy, and perceived psychological safety may influence pathways into care.

The results suggest that even a brief therapist response can shape proximal appraisals that are relevant to mental health engagement. The validation-based response increased perceived recognition and reduced autonomy-related resistance, and these appraisal pathways were associated with more favorable anticipated alliance, therapist credibility, expected treatment benefit, and willingness to continue. These findings indicate that therapist communication may influence engagement through two complementary behavioral and psychological mechanisms: strengthening the person’s sense of being understood and treated as legitimate, while reducing the likelihood that the encounter will be experienced as controlling, pressuring, or freedom-threatening.

These processes are relevant to health promotion because psychotherapy can only be beneficial when individuals are willing to enter, remain in, and collaborate within treatment. Responses that foster recognition and preserve autonomy may support early engagement, whereas responses that are experienced as imposing or autonomy-threatening may contribute to resistance, hesitation, or reduced willingness to continue. This may be especially important in community and public health contexts in which improving access to mental health care is not sufficient on its own. Individuals must also experience the first points of contact with psychological care as respectful, credible, and responsive to their agency.

The findings therefore point to the importance of communication practices that promote behavioral and mental health engagement. Training therapists and other mental health professionals to combine validation, recognition, and autonomy support with clinically necessary challenge may help reduce early resistance and strengthen motivation for continued care. More broadly, the study contributes to a prevention-oriented understanding of mental health engagement by identifying interpersonal conditions that may either facilitate or hinder the development of trust, collaboration, and willingness to pursue psychological help.

Although the study was conducted among Hebrew-speaking adults in Israel, the processes examined here are not limited to a specific national context. Early psychotherapy encounters, perceived recognition, autonomy-related resistance, and expectations regarding treatment benefit are relevant across diverse societies and service settings. The present findings therefore contribute to a broader understanding of how social and behavioral determinants within mental health encounters may influence engagement, wellbeing, and the promotion of effective psychological care.

### 4.2. Implications for Public Mental Health Services and Early Engagement

The present findings have implications for public mental health services, especially for the earliest points of contact between potential patients and psychological care. From a service-level perspective, early therapist communication should be treated as part of engagement-oriented mental health care rather than merely as an individual therapist preference or clinical style. Very brief therapist responses may shape whether potential patients experience psychological care as respectful, credible, autonomy-supportive, and worth continuing. These impressions are not limited to global liking or disliking of the therapist; they include appraisals of whether the therapist recognizes the person’s experience and whether the response preserves or threatens the person’s sense of agency. A validation-based response may support engagement by allowing the person to feel seen, understood, and treated as legitimate. A directive and challenging response may undermine early engagement when it feels evaluative, imposing, or autonomy-limiting. Thus, communication practices that promote recognition and preserve autonomy may be relevant not only to individual clinical process, but also to treatment uptake, early retention, and access to effective psychological care.

Second, the findings suggest that validation and challenge should not be treated as competing therapeutic stances. Challenge may be clinically necessary, especially when maladaptive interpersonal patterns need to be examined directly. However, the present findings suggest that challenge may be most useful when it is embedded within a relational frame of recognition, respect, collaboration, and autonomy support. The critical engagement issue is therefore not simply whether therapists should validate or challenge, but whether challenge is delivered in a way that preserves dignity, agency, and the possibility of collaboration.

Third, the findings are relevant for work with narcissistic features in mental health services. Prior research suggests that narcissistic pathology may complicate psychotherapy utilization, early treatment change, dropout, and the development of the therapeutic alliance [16,17,18]. Therapist-side research also suggests that narcissistic personality pathology can evoke distinctive countertransference patterns, including more hostile, criticized, helpless, and disengaged therapist responses [20]. The present findings do not suggest that narcissistic traits uniformly undermine psychotherapy engagement. Instead, they suggest that rivalry-related defensiveness may increase vulnerability to experiencing therapist responses as insufficiently recognizing or autonomy-threatening. Engagement-oriented training and supervision may therefore need to emphasize how interpretations, challenges, and comments about responsibility are likely to be heard by individuals high in rivalry.

At the same time, the unexpected positive residual association between rivalry and willingness to continue therapy suggests that such individuals should not be viewed simply as disengaged or treatment-avoidant. They may resist feeling controlled, judged, or diminished, but they may still be willing to continue when therapy feels serious, relevant, and potentially useful. This distinction has important implications for early engagement. The therapeutic task may not be to avoid difficult material, but to introduce it in a way that preserves recognition and autonomy while still allowing meaningful self-examination. For individuals higher in admiration, recognition and respect may support early receptivity to care, but engagement-oriented training and supervision should also emphasize that recognition is not equivalent to simple affirmation or collusion with grandiose self-views.

At a service level, these findings also suggest concrete points for implementation in public mental health systems. Intake procedures, first-session training, supervision, and brief-contact protocols may benefit from emphasizing communication that combines clear clinical direction with explicit recognition and autonomy support. Such practices may be especially important in settings where early dropout, hesitation to seek help, and low trust in services limit the public health impact of available psychological interventions. Future implementation work should examine whether recognition-supportive and autonomy-supportive communication during intake and first contacts improves actual treatment initiation, first-session attendance, early retention, and continuity of care.

### 4.3. Methodological Implications

The study also contributes methodologically to psychotherapy-process research. Vignette-based studies cannot replace research on actual therapy sessions, but they allow researchers to isolate specific interpersonal features of therapist response style while holding the clinical scenario constant. The present design shows that participants can form meaningful anticipatory judgments from brief therapist-response materials. This is consistent with prior analogue work showing that therapist warmth and competence can influence outcome expectations and alliance [5], and with broader evidence that therapist verbal interventions are meaningfully related to alliance and outcome-relevant processes [6]. The present study extends this work by identifying perceived recognition and autonomy-related resistance as proximal appraisal mechanisms that may help explain how early therapist responses shape anticipated engagement.

### 4.4. Limitations

Several limitations should be noted. First, the study used a vignette-based analogue design rather than actual psychotherapy sessions or naturally occurring contacts with mental health services. Participants imagined themselves in an early therapeutic encounter, but they were not responding to an actual therapist in an ongoing treatment relationship and were not making real decisions about service use. Therefore, the findings concern anticipated appraisals, expectations, and willingness to continue, not actual therapeutic alliance, treatment uptake, first-session attendance, dropout, treatment adherence, or clinical outcome. This distinction is important because analogue and highly controlled designs may not reproduce the experiential conditions of routine clinical practice, where distress, help-seeking, dependence, and the real possibility of continuing or discontinuing care shape how patients evaluate therapist responses [37,38,39,40]. Similarly, treatment expectations and autonomous motivation for therapy are not merely abstract attitudes toward hypothetical scenarios, but context-sensitive processes that are formed in relation to actual suffering, help-seeking, and treatment participation [12,27,28,39]. The public mental health implications should therefore be interpreted as implications for anticipated early engagement processes that require confirmation in real-world service settings, clinical samples, and naturally occurring first contacts with psychological care.

A related limitation concerns the activation of narcissistic self-regulatory dynamics. Narcissistic admiration and rivalry were assessed as trait dimensions, but the vignette was completed online, privately, and without interaction with an actual therapist. As a result, the situational cues that may activate narcissistic defensiveness, such as real interpersonal exposure, evaluation, dependence, shame, status threat, or the possibility of rupture, were likely attenuated. This may help explain why admiration and rivalry operated as general appraisal tendencies rather than as moderators of the therapist-response manipulation. In actual therapeutic relationships, narcissistic dynamics may become more strongly activated because patients are exposed to a real therapist, real evaluation, and real relational consequences [16,17,18,23]. The failure to support the moderation hypotheses should therefore be interpreted within the constraints of the analogue paradigm rather than as evidence that narcissistic traits cannot moderate responses to therapist behavior in clinical settings. Although the additional confirmatory analyses supported the discriminant validity of perceived recognition relative to anticipated therapeutic alliance, therapist credibility, and expected treatment benefit in the present dataset, they do not constitute full independent psychometric validation of the scale. Future research should validate the Perceived Recognition scale in independent samples, outside the present research group’s vignette-based paradigms, and across additional clinical, community, and service-based contexts.

Second, the sample consisted of Hebrew-speaking adults in Israel recruited through an online panel. Although this allowed for a large community sample, the findings may not generalize to clinical populations, to individuals currently seeking therapy, or to other cultural contexts. Third, the study examined a single clinical scenario and two carefully constructed therapist responses. Other presenting problems, therapeutic modalities, therapist identities, or forms of challenge may produce different patterns.

Fourth, all focal variables were assessed by self-report within a single assessment session. This design preserves temporal proximity to the stimulus, but it also raises concerns about shared method variance and does not allow conclusions about longer-term processes. Fifth, the study focused on narcissistic admiration and rivalry as dimensional aspects of grandiose narcissism. It did not assess vulnerable narcissism as a separate construct, nor did it diagnose narcissistic personality disorder. Thus, the results should be interpreted as evidence concerning narcissistic self-regulatory orientations in a community sample rather than as direct evidence about the treatment of diagnosed narcissistic personality pathology.

Sixth, although the two therapist responses were designed to differ primarily in interpersonal style and were validated by expert ratings and participant manipulation checks, therapist responses are necessarily complex. The directive and challenging response differed from the validation-based response not only in directiveness, but also in responsibility emphasis, interpretive certainty, and change-orientation. These elements are clinically intertwined, but future research should attempt to disentangle them more precisely.

Finally, although the study was framed in relation to public mental health engagement, the outcomes were analogous and anticipatory rather than behavioral indicators of actual service use, treatment uptake, or retention. Future research should examine whether recognition-supportive and autonomy-supportive communication predicts real-world help-seeking, first-session attendance, early dropout, and continued engagement across mental health service settings.

### 4.5. Future Directions

Future research should extend these findings in several directions. First, studies should examine whether similar recognition–resistance pathways emerge in actual clinical or semi-clinical settings, including simulated therapy sessions, standardized-patient designs, video-based therapist-response paradigms, intake encounters, and naturally occurring first sessions. Second, longitudinal research is needed to determine whether early perceived recognition and autonomy-related resistance predict actual alliance development, first-session attendance, dropout, session attendance, treatment satisfaction, continuity of care, or symptom change. Such studies would help determine whether the anticipated engagement processes observed here translate into real-world mental health service engagement and retention.

Third, future studies should test more nuanced therapist-response conditions, especially responses that combine validation with challenge and that vary the degree of therapist directness, structure, seriousness, expertise, and authority. Such designs would help determine whether the residual direct effects observed in the present models reflect meaningful features of challenge-based communication or model-dependent suppression. Fourth, future research should examine clinical samples, including individuals with elevated narcissistic traits, personality pathology, shame sensitivity, or difficulties with self-esteem regulation, in order to test whether narcissistic self-regulatory dynamics become stronger when real interpersonal exposure, vulnerability, and evaluative stakes are present.

Fifth, future studies should examine whether vulnerable narcissism, attachment insecurity, trait reactance, shame sensitivity, and client preference for therapist directiveness moderate responses to therapist style more strongly than admiration and rivalry did in the present study. Finally, future intervention-focused work should examine whether therapists can be trained to deliver challenging interventions in ways that preserve recognition and autonomy and whether such training improves early engagement, reduces rupture or early dropout, and strengthens continuity of care among patients who are prone to defensiveness or reactance.

## 5. Conclusions

The present study suggests that early therapist response style may shape anticipated engagement with psychological care through two proximal appraisal pathways: perceived recognition and autonomy-related resistance. A validation-based, recognition-supportive, autonomy-supportive therapist response elicited greater perceived recognition and lower autonomy-related resistance than a more directive and challenging response, and these appraisals were strongly associated with anticipated therapeutic alliance, therapist credibility, expected treatment benefit, and willingness to continue with the therapist. However, narcissistic admiration and rivalry did not moderate the experimental effects or the indirect pathways as predicted. Thus, the moderated-mediation component of the model should be regarded as unsupported in the present study and in need of independent replication before stronger conditional claims are made. More cautiously, the findings suggest that first-contact therapist communication may be a candidate modifiable feature of anticipated public mental health engagement, but its implications for actual treatment uptake, early retention, trust in services, and access to effective psychological care require confirmation in clinical samples, real service settings, and longitudinal designs.

## Figures and Tables

**Table 1:** Sociodemographic and background information.

		Men (*n* = 482)		Women (*n* = 490)		
		Experimental Condition				
	Total Sample (*N* = 972)	Validation-Based (*n* = 249)	Challenge-Based (*n* = 233)	Validation-Based (*n* = 247)	Challenge-Based (*n* = 243)	Validation-Based ≠ Challenge-Based
Age	45.12	46.10	46.53	42.66	45.25	*t* = −1.56
Familiarity with Psychotherapy	50.41	42.29	48.21	56.40	54.75	*t* = −0.99
Helpfulness of Psychotherapy	57.94	53.46	53.95	61.02	63.23	*t* = −0.72
Prior Psychotherapy						*χ*^2^ = 4.32
Yes	48.1%	41.4%	43.3%	61.5%	46.1%	
No	51.9%	58.6%	56.7%	38.5%	53.9%	
Current Psychotherapy						*χ*^2^ = 1.74
Yes	12.3%	10.4%	10.7%	17.0%	11.1%	
No	87.7%	89.6%	89.3%	83.0%	88.9%	
Number of Previous Therapists						*χ*^2^ = 3.45
None	50.7%	56.6%	55.4%	39.3%	51.9%	
One	23.4%	24.5%	21.0%	24.7%	23.0%	
Two	15.5%	12.9%	15.0%	19.0%	15.2%	
Three or more	10.4%	6.0%	8.6%	17.0%	9.9%	
Duration of Therapy						*χ*^2^ = 10.57
Never	50.3%	55.4%	55.4%	38.5%	52.3%	
Less than 3 months	11.8%	14.9%	10.3%	13.0%	9.1%	
3–6 months	10.8%	11.6%	13.7%	7.7%	10.3%	
6–12 months	7.1%	4.8%	6.0%	12.1%	5.3%	
1–2 years	7.9%	5.6%	6.0%	11.7%	8.2%	
More than 2 years	12.0%	7.6%	8.6%	17.0%	14.8%	
Previous Medication						*χ*^2^ = 0.12
Yes	13.1%	12.0%	15.5%	13.4%	11.5%	
No	86.9%	88.0%	84.5%	86.6%	88.5%	
Current Medication						*χ*^2^ = 0.01
Yes	9.6%	7.2%	10.7%	11.7%	8.6%	
No	90.4%	92.8%	89.3%	88.3%	91.4%	
Education						*χ*^2^ = 5.39
No high school degree	14.7%	17.7%	21.9%	8.5%	11.1%	
High school degree	24.0%	22.9%	24.5%	22.7%	25.9%	
Bachelor’s degree	39.2%	38.2%	35.2%	44.1%	39.1%	
Master’s degree	20.5%	18.9%	17.6%	22.7%	22.6%	
Ph.D. or equivalent	1.6%	2.4%	0.9%	2.0%	1.2%	
Employment						*χ*^2^ = 10.44
Full time	60.9%	61.0%	67.8%	70.3%	59.5%	
Part time	18.6%	12.9%	14.6%	15.8%	20.6%	
Unemployed	7.7%	8.4%	5.6%	2.5%	10.1%	
Going to school	1.6%	3.2%	1.7%	6.4%	0.8%	
Homemaker	8.6%	10.8%	8.6%	6.4%	6.1%	
Retired	2.5%	3.2%	3.4%	1.0%	1.6%	
Marital Status						*χ*^2^ = 9.81
Single	15.2%	10.8%	16.7%	15.4%	18.1%	
Dating	3.5%	4.4%	3.0%	4.8%	1.6%	
Cohabiting	5.1%	5.2%	3.0%	6.9%	5.3%	
Married	64.7%	71.5%	70.0%	58.3%	59.3%	
Separated	0.4%	0.4%	0.4%	0.4%	0.4%	
Divorced	9.8%	5.2%	6.9%	13.4%	13.6%	
Widowed	1.2%	2.4%	0.0%	0.8%	1.6%	
Household income						*χ*^2^ = 2.62
Very high	12.9%	17.7%	16.7%	7.3%	9.9%	
Somewhat high	24.2%	30.5%	24.9%	21.9%	19.3%	
Moderate	25.9%	23.7%	27.9%	25.5%	26.7%	
Somewhat low	22.0%	16.5%	18.9%	26.7%	25.9%	
Very low	15.0%	11.6%	11.6%	18.6%	18.1%	
Religiosity						*χ*^2^ = 1.33
Secular	54.7%	51.8%	58.8%	54.7%	53.9%	
Traditional	18.8%	18.5%	15.5%	21.1%	20.2%	
Religious	11.0%	13.3%	7.3%	10.1%	13.2%	
Ultra-Orthodox	15.4%	16.5%	18.5%	14.2%	12.8%	

**Table 2:** (**A**) Descriptive statistics by experimental condition. (**B**) Zero-order correlations among the study variables by experimental condition.

(**A**)								
	**Validation-Based** **Condition (*n* = 496)**				**Challenge-Based** **Condition (*n* = 476)**			
	***M***	***SD***	***Skewness***	***Kurtosis***	***M***	***SD***	***Skewness***	***Kurtosis***
1. Narcissistic Admiration	3.57	0.82	0.04	−0.30	3.53	0.86	−0.05	−0.27
2. Narcissistic Rivalry	2.16	0.77	0.77	0.20	2.10	0.77	0.81	0.24
3. Perceived Recognition	5.28	1.24	−0.61	−0.07	4.75	1.30	−0.24	−0.33
4. Autonomy-Related Resistance	−0.29	0.80	0.56	−0.65	0.26	0.84	0.33	−0.48
5. Anticipated Therapeutic Alliance	3.57	0.65	−0.31	0.07	3.49	0.69	−0.39	0.37
6. Therapist Credibility	6.01	1.68	−0.38	−0.11	5.97	1.80	−0.38	−0.43
7. Expected Treatment Benefit	0.04	0.84	−0.43	−0.16	0.02	0.90	−0.44	−0.26
8. Willingness to Continue Therapy	3.71	1.05	−0.68	−0.02	3.48	1.08	−0.41	−0.50
(**B**)								
	**1**	**2**	**3**	**4**	**5**	**6**	**7**	**8**
1. Narcissistic Admiration	–	0.08	0.12 **	0.03	0.13 **	0.12 *	0.16 ***	0.13 **
2. Narcissistic Rivalry	0.11 *	–	−0.09	0.26 ***	−0.16 ***	−0.11 *	−0.13 **	−0.03
3. Perceived Recognition	0.08	−0.10 *	–	−0.45 ***	0.65 ***	0.55 ***	0.49 ***	0.61 ***
4. Autonomy-Related Resistance	0.03	0.27 ***	−0.59 ***	–	−0.52 ***	−0.45 ***	−0.41 ***	−0.43 ***
5. Anticipated Therapeutic Alliance	0.13 **	−0.08	0.66 ***	−0.53 ***	–	0.68 ***	0.67 ***	0.78 ***
6. Therapist Credibility	0.11 *	−0.04	0.61 ***	−0.53 ***	0.70 ***	–	0.73 ***	0.68 ***
7. Expected Treatment Benefit	0.14 **	−0.07	0.50 ***	−0.37 ***	0.64 ***	0.58 ***	–	0.65 ***
8. Willingness to Continue Therapy	0.06	−0.07	0.65 ***	−0.57 ***	0.78 ***	0.71 ***	0.61 ***	–

*Note*. Validation-based and challenge-based condition statistics are presented separately for each study variable. *M* = mean; *SD* = standard deviation. The values below the diagonal are taken from participants in the validation-based condition, whereas the values above the diagonal are taken from participants in the challenge-based condition. * *p* < 0.05; ** *p* < 0.01; *** *p* < 0.001.

**Table 3:** Comparisons of the validation-based and challenge-based conditions.

	Validation-Based Condition (*n* = 496)		Challenge-Based Condition (*n* = 476)			
	*M*	*SD*	*M*	*SD*	*t*_(970)_	*p*
Narcissistic Admiration	3.57	0.82	3.53	0.86	0.62	0.537
Narcissistic Rivalry	2.16	0.77	2.10	0.77	1.17	0.241
Perceived Recognition	5.28	1.24	4.74	1.30	6.60	<0.001
Autonomy-Related Resistance	−0.29	0.80	0.26	0.84	−10.44	<0.001
Anticipated Therapeutic Alliance	3.57	0.65	3.49	0.69	1.84	0.066
Therapist Credibility	6.01	1.68	5.97	1.80	0.32	0.746
Expected Treatment Benefit	0.04	0.84	0.02	0.90	0.38	0.704
Willingness to Continue Therapy	3.71	1.05	3.48	1.08	3.27	0.001

*Note*. Group differences were tested using two-tailed independent-samples *t* tests.

## Data Availability

The preregistration, study materials, and anonymized SPSS dataset used for the reported analyses are openly available on the Open Science Framework (OSF) at https://osf.io/asq42, accessed on 1 July 2026.
